# Assessing the Relationship between Performance on the University of California Performance Skills Assessment (UPSA) and Outcomes in Schizophrenia: A Systematic Review and Evidence Synthesis

**DOI:** 10.1155/2018/9075174

**Published:** 2018-12-27

**Authors:** Shelagh Szabo, Elizabeth Merikle, Greta Lozano-Ortega, Lauren Powell, Thomas Macek, Stephanie Cline

**Affiliations:** ^1^Broadstreet HEOR, Vancouver, BC, Canada; ^2^Takeda Pharmaceuticals, Deerfield, IL, USA

## Abstract

**Objective:**

To perform a systematic review of the published literature to evaluate how functional capacity, as measured by the University of California at San Diego (UCSD) Performance-based Skills Assessment (UPSA), relates to other functional measures and real-world outcomes among individuals with schizophrenia.

**Methods:**

The MEDLINE® and Embase® databases were searched to identify joint evaluations with UPSA and key functional outcomes (functional scale measures; generic or disease-specific, health-related quality of life [HRQoL]; or real-world outcomes [residential status; employment status]) in patients with schizophrenia. Pearson correlations were estimated between UPSA scores, HRQoL, other functional scale measures, and real-world outcomes, for outcomes described in at least six studies.

**Results:**

The synthesis included 76 studies that provided 73 unique data sets. Quantitative assessment between the Specific Level of Function (SLOF) (n=18) scores and UPSA scores demonstrated a moderate borderline-significant correlation (0.45,* p*=0.06). Quantitative analysis of the relationship between the Global Assessment of Functioning (GAF) (n=11) and the Multidimensional Scale of Independent Functioning (MSIF) (n=6) scales revealed moderate and small nonsignificant Pearson correlations of -0.34 (*p=*0.31) and 0.12 (*p=*0.83), respectively. There was a small borderline-significant correlation between UPSA score and residential status (n=36; 0.31;* p=*0.08), while no correlation was found between UPSA score and employment status (n=19; 0.04;* p*=0.88).

**Conclusion:**

The SLOF was the most often used functional measure and had the strongest observed correlation with the UPSA. Although knowledge gaps remain, evidence from this review indicates that there is a quantitative relationship between functional capacity and real-world outcomes in individuals with schizophrenia.

## 1. Introduction

Cognitive dysfunction has been recognized as a core feature of schizophrenia and an important determinant of health outcomes [1, 2]. The cognitive impacts of schizophrenia tend to be present at illness onset, remain relatively stable over time, and have a neutral response to the antipsychotic medications that are effective at treating other symptoms of schizophrenia [3–7]. Individual domains of cognitive ability (including learning, attention, and executive functioning), and composite scores on measures of these, are correlated with everyday functioning [8, 9]. As a result, cognitive dysfunction is thought to be a substantial contributor to the functional disability associated with schizophrenia.

It is not surprising, therefore, that, with the recent shift in the management of schizophrenia from symptom control to functional recovery, pharmacotherapies developed to treat cognition in schizophrenia are expected to demonstrate improvement on both cognitive and functional co-primary endpoints [2]. The psychometric characteristics and practicality of various performance-based measures of functional capacity in patients with schizophrenia have been assessed in the Measurement and Treatment Research to Improve Cognition in Schizophrenia (MATRICS) Validation of Intermediate Measures (VIM) study [2]. It concluded that the University of California at San Diego (UCSD) Performance-based Skills Assessment (UPSA) was the superior co-primary measure to be included in randomized trials [2]. The UPSA measures capacity in five domains of functioning, including household chores, communication, finance, transportation, and planning recreational activities, by scoring patients as they complete a series of simulated daily activities in a clinical setting [10]. UPSA performance is significantly impaired among individuals with schizophrenia or schizoaffective disorder compared with healthy controls [10] and is able to predict real-world functional outcomes [11, 12].

The UPSA is considered the standard performance-based measure of functional capacity among individuals with schizophrenia, as it provides a fair balance between ease of administration, reliability, and validity in relation to real-world functional outcomes [11, 12]. However, the strength of the relationship between the UPSA and real-world functional outcomes, and how it applies across the various functional measures used to measure treatment efficacy and effectiveness in schizophrenia, is not clear. Therefore, the objective of this study was to synthesize published evidence on how functional capacity, as measured by the UPSA, relates to other functional measures and real-world outcomes among individuals with schizophrenia. The findings from this study will highlight the strength of the evidence of the relationship between measures of functional capacity and functional outcomes in schizophrenia and identify knowledge gaps.

## 2. Methods

A systematic review of the published literature was conducted to identify studies in individuals diagnosed with schizophrenia on whom joint evaluations of functional capacity, measured using the UPSA, and* a priori*-determined key functional outcomes (functional scale measures; generic or disease-specific, health-related quality of life (HRQoL); employment status; residential status) were performed.

### 2.1. Search Strategy

The MEDLINE® and Embase® databases were searched using terms related to schizophrenia, the UPSA, and generic and specific functional measures and outcomes. Studies that matched the predetermined criteria according to a PICOS (Population, Intervention/Comparators, Outcomes, Study) approach (Table S1) were included in this review. Other evidence synthesis or decision-modeling studies or case reports were excluded. The literature review was limited to publications in English from 2000 to April 27, 2017, with a minimum sample size of ten participants.

### 2.2. Study Selection

Two researchers independently reviewed all abstracts identified by the search strategy against the PICOS criteria and then reviewed the full text of all potentially relevant abstracts. Discrepancies between the studies selected for inclusion by the two researchers were arbitrated by a third researcher.

### 2.3. Data Extraction

Study design, baseline clinical and demographic characteristics of the patient populations, and outcomes data of interest from the eligible studies were extracted into Excel®. Outcomes data included scores on the UPSA, HRQoL, other functional scale measures, and real-world outcomes such as residential and employment status. As the objective was to understand how the range of scores on each functional measure related to the range of UPSA scores, the focus was on measures where > two studies reported outcomes using that measure; measures with ≤ two sets of scores would not allow for meaningful comparison.

Where available, both baseline and final scores on functional measures were extracted, as well as changes in scores over time. For continuous variables, the mean, median, standard deviation, and range were extracted; for dichotomous and categorical variables, the number of patients and proportion were extracted.

### 2.4. Quality of Included Studies

The quality of the included studies was assessed using the Strengthening of Reporting in Observational Studies (STROBE) checklist (http://strobe-statement.org/index.php?id=available-checklists).

### 2.5. Statistical Analysis

The synthesis focused on the functional measures most frequently described in association with UPSA scores. For functional measures described in at least six studies, scatter plots of overall UPSA score versus the score on the functional measure were created. Pearson correlations were estimated between the functional measure score and the UPSA score: overall, and according to the type of UPSA used (full UPSA versus UPSA-Brief [UPSA-B]). These were estimated in R version 3.4.0, via the* cor.test* function, which also reported the statistical significance of the correlation coefficient. In this analysis, p values of 0.05 or lower were considered statistically significant, while those greater than 0.05 but lower than 0.1 were considered borderline statistically significant. Regardless of statistical significance, each correlation coefficient calculated was categorized as small (0.1-0.3), moderate (0.3-0.5), and large (>0.5) [13]. For measures described in ≤ five studies, associations with the UPSA were qualitatively described. Overall UPSA scores were imputed in studies that only reported subdomain UPSA scores or provided raw scores.

To evaluate real-world outcomes, the relationship between UPSA scores and current living situation or current employment status was assessed. Current living situation as presented by the original articles was recategorized into four groups based on level of care and supervision required: (1) living independently, defined as individuals residing in the community (alone, with a roommate, or with their family); (2) community-dwelling assisted living, defined as individuals residing in sheltered housing, board-and-care homes, and residential care homes; (3) restricted living, defined as individuals residing in locked board-and-care homes and restricted housing; and (4) institutionalized, defined as individuals being cared for in skilled-nursing homes and psychiatric hospitals. Current employment status, also presented by the original articles, was recategorized into three distinct groupings: (1) not employed; (2) employed, including full- or part-time paid employment; and (3) other, such as volunteers, students, sheltered employment, retired, and people on disability.

## 3. Results

**Figure 1:**
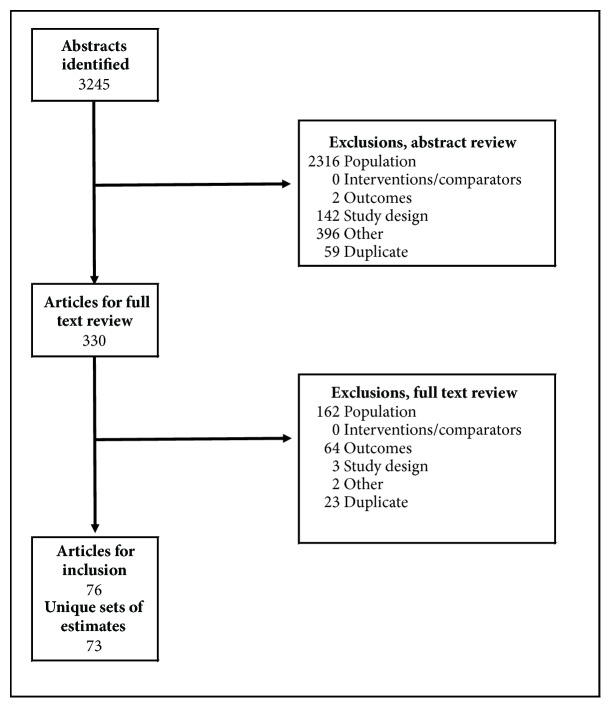
Flow chart of article screening and selection process.

The searches identified 3,245 articles. Titles and abstracts were screened, and 330 studies were considered potentially eligible for inclusion. Full-text articles were retrieved. After analyzing the full-text articles, 254 studies were excluded and 76 studies were found eligible for inclusion according to our criteria for considering studies in this review. These 76 studies provided 73 unique sets of data (subsequently referred to as studies) [2, 10, 14–84], because three of the studies presented evidence from samples already described in other included publications (Figure 1).

All 73 included studies reported the UPSA, 25 (34%) included studies reported the Specific Level of Function (SLOF), and >5% of the included studies reported the Global Assessment of Functioning (GAF), the Quality of Life Scale (QLS), the Multidimensional Scale of Independent Functioning (MSIF), and the Quality of Life Interview (QOLI) (Table 1). A brief description of the scales is presented in Table S2. Of the 73 included studies, 41 reported the full UPSA, 33 reported the UPSA-B, two reported the UPSA version 2 (UPSA-2), one reported the UPSA tablet/mobile application (UPSA-M) and UPSA-M-Brief, one reported the Computerized UPSA (C-UPSA), and one reported the UPSA-VIM. Only the full UPSA and UPSA-B provided a large enough evidence base (i.e., were reported on by ≥ six studies) for a quantitative assessment of the relationship between the UPSA and other functional measures, with a total of 68 studies reporting either of those measures, or both.

Characteristics of the included studies are presented in Table S3, and baseline characteristics of the patient populations in the included studies are presented in Table 2.

### 3.1. Study Quality Assessment

The quality assessment of the included studies was conducted according to the STROBE statement recommendation for reporting in observational studies (Table S4). Overall, the included studies had clearly defined objectives and presented detailed results of both primary and secondary objectives. They provided data sources as well as methods of assessment for each outcome reported. However, potential sources of bias were poorly reported, with only 9 out of the 73 studies addressing bias and any efforts to minimize it. Moreover, none of the included studies described how the study size was determined or whether power calculations were performed. Less than half (45%) of studies discussed the generalizability of the findings outside of the population investigated.

### 3.2. Quantitative Assessment of the Relationship between the UPSA and Other Functional Measures

#### 3.2.1. UPSA versus SLOF

The UPSA and the SLOF were jointly evaluated in 25 studies. Quantitative assessment of the correlation between the UPSA and the SLOF was based on estimates from 18 studies, which reported the overall SLOF score, or enough information to derive it [17, 18, 20–24, 26–30, 34, 36, 56, 76, 79]. Of these, four studies reported the full length UPSA [20, 21, 24, 34], 13 studies reported the UPSA-B [17, 18, 22, 24, 26–30, 36, 56, 76], and one study reported the UPSA-VIM [79]. Baseline characteristics of the patient populations in these studies with respect to gender, age, ethnicity, and years of education were similar. Information on schizophrenia diagnosis (as opposed to schizoaffective disorder), antipsychotic use, living independently, and employment status was limited.

**Figure 2:**
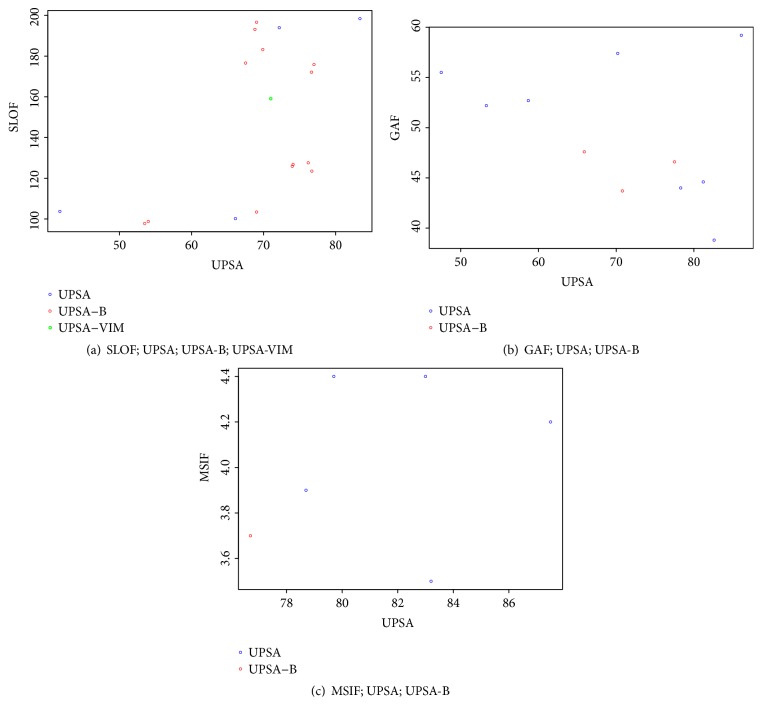
Scatterplot of UPSA and scores on functional measures from studies that reported both measures.

Quantitative analysis revealed a moderate, positive, borderline-significant Pearson correlation across these 18 studies of 0.45 (*p*=0.06) (Figure 2(a); Table S5).

#### 3.2.2. UPSA versus GAF

The UPSA and the GAF were jointly evaluated in 11 studies [35, 37, 39–45, 47, 60]. Quantitative assessment of the correlation between the UPSA and the GAF was based on 11 pairs of estimates from nine studies, which reported the overall UPSA score, or enough information to derive it. Of these, six studies reported the UPSA [37, 40, 42, 43, 47, 60], and three studies reported the UPSA-B [35, 41, 45]. Baseline characteristics of the patient populations in these studies showed that the majority of studies included more males than females; two studies had samples with an average age < 30 years, while the rest of the studies had a mean age of ≥ 40 years. The proportion of patients with a schizophrenia diagnosis was comparable across studies. Information on ethnicity, years of education, antipsychotic use, and residential and employment status was limited.

Quantitative analysis demonstrated a moderate, negative, and nonsignificant Pearson correlation across all studies of -0.34 (*p=*0.31) (Figure 2(b); Table S6).

#### 3.2.3. UPSA versus MSIF

The UPSA and the MSIF were jointly evaluated in six studies [52–57]. Quantitative assessment of the correlation between the UPSA and the MSIF was based on estimates from all six studies. Of these, five studies reported the UPSA [52–55, 57], and one study reported the UPSA-B [56]. Baseline characteristics of the patient populations in these studies with respect to gender and age were similar. Information on all other baseline characteristics was limited.

Quantitative analysis revealed a small nonsignificant Pearson correlation across all studies of 0.12 (*p=*0.83) (Figure 2(c); Table S7).

### 3.3. Qualitative Assessment of the Relationship between the UPSA and Other Functional Measures

Associations of the UPSA with functional measures reported in five or fewer studies were qualitatively described based on the authors' considerations and findings. The UPSA and the QLS were jointly evaluated in seven studies [2, 39, 47, 58, 63, 76, 77]. Of these, four reported the full UPSA [2, 47, 58, 63], and three reported the UPSA-B [39, 76, 77]. Two studies could not be incorporated in a quantitative assessment of the relationship between the UPSA and the QLS, as one study reported a raw UPSA-B score [39] and one study did not report an overall UPSA score or information from which one could be derived [58]. Five studies reported correlations between UPSA and the QLS scores ranging from 0.15 to 0.29 [2, 47, 63, 76, 77].

The UPSA-B and the Strauss-Carpenter scale were jointly evaluated in two studies [35, 61]. One study reported a correlation between UPSA-B score and areas of housing, ability to work, and social contacts measured with the Strauss-Carpenter scale [35]. The UPSA and the Personal and Social Performance Scale (PSP) were jointly evaluated in two studies [37, 39]. One study reported a correlation between the two scales of 0.42 (*p*<0.0001) [37]. The full or brief UPSA and the Role Functioning Scale (RFS) were jointly evaluated in four studies [41, 45, 81, 83]. Only one study assessed the correlation between the two scales [83], reporting a value of 0.47. The full UPSA and the Independent Living Skills Survey (ILSS) were jointly evaluated in two studies [46, 47]. One study reported a correlation between the two scales of 0.13 (*p*=0.24) and concluded that the UPSA did not correlate well with the ILSS [46]. The other study reported a correlation of 0.16 (*p*=0.28) [47]. The UPSA and the Social Functioning Scale (SFS) were jointly evaluated in three studies [60, 76, 80], two of which assessed the correlation between the two scales. One study reported a correlation between full UPSA and SFS scores of 0.29. The authors suggested that within-site community functioning homogeneity resulted in variability in the size of correlations across sites [60]. The other study reported a correlation between UPSA-B and SFS scores of 0.10 (self-reported SFS;* p*>0.05) and 0.24 (proxy SPS:* p*< 0.05) [76]. The full UPSA and the Quality of Well-being Scale (QWB) were jointly evaluated in two studies [10, 74]. Only one assessed the correlation between the two scales [10], reporting a value of 0.28 (*p*>0.05). The UPSA and the Independent Living Skills Inventory (ILSI) were jointly evaluated in one study, which reported a correlation between the two scales of 0.40 (*p*<0.001) [64]. The UPSA and the Life Skills Profile (LSP) were jointly evaluated in one study, which reported a correlation between the two scales of 0.07 (self-reported) and 0.08 (proxy) (*p*>0.05) [76]. The UPSA and the Social Behavior Scale (SBS) were jointly evaluated in one study, which reported a correlation between the two scales of 0.06 (self-reported) and 0.10 (proxy) (*p*>0.05) [76]. The UPSA and the Medical Outcomes Survey–Short-form 36 (SF-36) were jointly evaluated in one study, which reported a correlation between the full UPSA and SF-36 scores of 0.1 (*p*>0.05) [72].

### 3.4. Quantitative Assessment of the Relationship between the UPSA and Real-World Outcomes

#### 3.4.1. UPSA versus Residential Status from Demographic Characteristics of the Study Sample

Quantitative assessment of the correlation between the UPSA and residential status at baseline incorporated estimates reported in 36 studies [10, 15, 16, 19, 23, 24, 29, 32, 34, 35, 37, 42, 45, 48–51, 59, 61–72, 76, 78, 80, 82, 84]. Of these, 20 studies reported the UPSA [10, 15, 19, 23, 32, 34, 37, 42, 48–51, 62–64, 69, 71, 72, 82, 84], 15 studies reported the UPSA-B [16, 24, 29, 35, 45, 59, 61, 65–67, 76, 78, 80, 82], and one study reported the UPSA-2 [70]. Baseline characteristics of the patient populations in these studies with respect to age, schizophrenia diagnosis, years of education, proportion using antipsychotics, and proportion of Caucasian participants were similar, when reported. Information on employment status was limited and variable.

**Figure 3:**
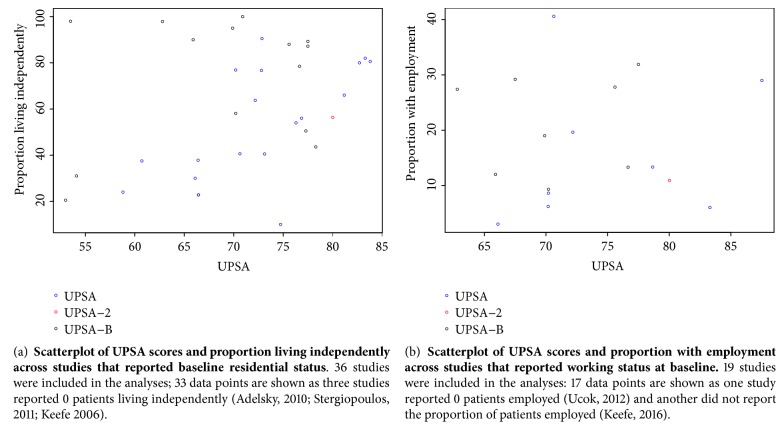
UPSA vs residential and employment status.

Quantitative analysis revealed a moderate borderline-significant correlation between UPSA score and residential status, as described by the proportion of patients living independently, across all studies (0.31;* p=*0.08) (Figure 3(a); Table S8). There was a large, significant correlation between full UPSA score and the proportion of patients living independently (0.65;* p*<0.01), but a small, nonsignificant correlation between UPSA-B score and proportion of patients living independently (0.26;* p*=0.37).

#### 3.4.2. UPSA versus Employment Status from Demographic Characteristics of the Study Sample

Quantitative assessment of the correlation between the UPSA and employment status at baseline incorporated estimates reported in 19 studies [16, 19, 23, 26, 29, 34, 35, 37, 39, 50, 52, 55, 61, 66, 70, 71, 76, 78, 79]. Of these studies, eight reported the UPSA [19, 23, 34, 37, 50, 52, 55, 71], nine the UPSA-B [16, 26, 29, 35, 39, 61, 66, 76, 78], one the UPSA-2 [70], and one the UPSA-VIM [79]. Baseline characteristics of the patient populations in these studies with respect to age, gender, ethnicity, and schizophrenia diagnosis were similar, when reported.

Quantitative analysis revealed no correlation between UPSA score and employment status, as described by the proportion of employed patients, across all studies (0.04;* p*=0.88) (Figure 3(b); Table S9). There was a small nonsignificant correlation between full UPSA score and proportion with employment 0.22 (*p*=0.60) and a small nonsignificant correlation between UPSA-B score and proportion with employment 0.11 (*p*=0.80).

### 3.5. Qualitative Assessment of the Relationship between the UPSA and Real-World Outcomes

#### 3.5.1. Residential Status as an Outcome

Seven studies investigated the correlation of the UPSA with current living situation [15, 16, 42, 49, 66, 67, 71]. Some findings supported the utility of the UPSA as a proxy for assessing real-world functioning, reporting correlations between UPSA total score and degree of independence in community living of 0.48 (*p*=0.001) and 0.44 (*p*<0.05) [15, 42]. Other studies concluded that UPSA score did not significantly correlate with independent living. One study reported a correlation of 0.21 that was not statistically significant [67]. Another study included homeless and housed individuals with schizophrenia; there was no significant difference between housed and homeless groups in total UPSA score, which may have been due to small sample size [71]. Two studies indicated that the UPSA-B was useful for predicting residential status among individuals with schizophrenia based on regression analyses that showed a significant relationship [16, 66]. One study indicated that the UPSA was significantly better than chance and better than classical clinical features of schizophrenia (e.g., positive and negative symptoms and global cognitive functioning) at predicting residential independence [49].

#### 3.5.2. Employment Status as an Outcome

Five studies investigated the correlation of the UPSA with employment status [16, 66, 67, 72, 73]. Two studies estimated correlations of the UPSA with employment status [72, 73]. One reported correlations between UPSA total score and attainment of competitive work, weeks of competitive work, and wages from competitive work at 0.04 to 0.10; none of these correlations were statistically significant [73]. The other reported a correlation between UPSA score and employment status at 0.19 (*p*<0.01) [72]. Three studies investigated the use of the UPSA-B for predicting employment status [16, 66, 67]. One reported a correlation between UPSA-B score and number of hours worked per week at 0.43 (*p*=0.001), concluding that being employed did not correlate with the UPSA-B, but hours of employment per week did [16]. Another reported a correlation between UPSA-B score and employment status of 0.09 (*p*>0.05) [67].

**Figure 4:**
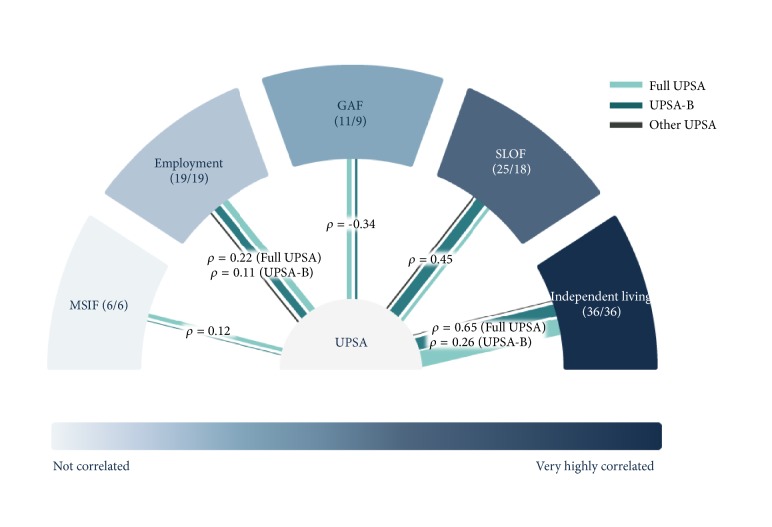
**The strength of correlations between UPSA and employment status, residential status, the SLOF, MSIF, and GAF. **Strength of correlations indicated by a correlation coefficient (according to UPSA type, if sufficient data are available) and color. The number of studies contributing evidence to a particular relationship is indicated by the thickness of the line connecting the UPSA to each of the other measures.

The strength of the relationships between the measures examined quantitatively (employment status, residential status, the SLOF, GAF, and MSIF) and UPSA is depicted in Figure 4, stratified according to UPSA type.

## 4. Discussion

This systematic literature review and evidence synthesis from 73 articles evaluated how functional capacity, as measured by the UPSA, relates to other functional measures and real-world outcomes among individuals with schizophrenia. Understanding this relationship is critical to determine the usefulness of performance on functional capacity measures as potential outcome measures in schizophrenia clinical trials.

Correlations between the UPSA and functional measures were estimated where at least six studies jointly evaluated both measures. Sufficient evidence to assess correlation with the UPSA, across and within studies, was only available for the SLOF, the GAF, and the MSIF. With respect to the SLOF, the current study provided evidence of a moderate correlation with the UPSA (*ρ*=0.45;* p*=0.06). This is consistent with findings from the individual studies that reported on this relationship, with correlations that were statistically significant, ranging from 0.19 [76] to 0.57 [36]. Some of the studies that investigated the correlation between the UPSA and the SLOF explored differences between the self-reported and proxy-reported SLOF and indicated that the UPSA correlated with the SLOF-proxy (particularly when the SLOF was reported by the clinician), but not with the self-reported SLOF [25, 76]. Based on these findings, we focused on the SLOF reported by proxy, which was used most frequently in the investigations of association between the UPSA and the SLOF, to avoid incorporation of heterogeneity in the estimates.

For the GAF, the MSIF, the QLS, the Strauss-Carpenter, the PSP, and the ILSI, there was evidence from study authors' assessments that these functional measures do correlate with the UPSA, although the current study was not able to demonstrate similar findings when grouping results. Of these measures, the GAF and the QLS showed the strongest correlations, which were consistently reported in two [35, 42] and five [2, 47, 58, 76, 77] studies, respectively. In the current study, the correlation between the UPSA and the GAF was not statistically significant, and in the opposite direction of the expected association. This counterintuitive estimate may have resulted from study heterogeneity or sparsity of evidence. However, the high degree of underreporting of baseline characteristics other than age and gender did not enable an accurate assessment of study heterogeneity.

The correlation of the UPSA with real-world functional outcomes, specifically the ability to live independently and to work, was investigated. The UPSA, particularly the full UPSA versus the UPSA-B, correlated well with residential status, specifically the proportion of individuals living independently. In contrast, authors of individual studies using the UPSA-B found good correlations with residential status [16]. Undetected heterogeneity may explain why this relationship was not observed when aggregating across studies.

The current study found no association between the UPSA and ability to work. These findings were consistent with those reported by other authors who have investigated these relationships within their own studies. Interestingly, one study found a moderate correlation between the UPSA and hours worked per week (*ρ*=0.43;* p*=0.001) and concluded that while hours of employment correlated well with the UPSA-B, being employed did not [16]. Authors of these studies did not provide further comment regarding the lack of association between the UPSA and ability to work.

While the available data were limited, the observed correlations between the UPSA, most functional measures, and residential status support the value of use of assessments of functional capacity to track functional status among patients in trials of schizophrenia treatments. This is important because although there are limitations to the use of functional simulation measures like the UPSA, they were developed, at least in part, to address challenges inherent in other measures of functional status for measuring outcomes in schizophrenia trials. For example, changes in real-world community functioning would be very compelling, but are unlikely to be observed over the course of a randomized trial in response to a particular treatment [2, 85]. Interviewer-based measures can also be limited as many behaviours cannot naturally be observed under such settings. Similarly, self-report can be unreliable among members of many patient groups, including among those with schizophrenia; and many schizophrenia sufferers do not have close informants to provide proxy reports [11]. Additionally, the different measures of functional status for schizophrenia all include slightly different combinations of constructs within their measure; this may in part affect the degree of any relationship observed with performance on the UPSA.

Of course, there are situations where a real-world outcome or another functional measure may be most appropriate compared to the UPSA; and ultimately, measure selection should be driven by a number of factors including trial/study duration, the severity of the patient population included, and the need for comparability of the study findings with the results of other studies.

## 5. Limitations

A key strength of the current study is that a comprehensive, systematic approach to identifying and synthesizing all relevant articles where the UPSA was evaluated along with another functional measure was undertaken. Estimating correlations based on data presented for a particular measure across a number of different studies is a novel approach that aims to fill a gap in the current knowledge regarding how well assessments of functional capacity in schizophrenia correlate with other functional measures and real-world functional outcomes. However, this study was associated with some limitations. First, as with any systematic review, the findings were limited by the heterogeneity in the design, validity, and reporting of the studies contributing estimates. Second, several studies by the same study groups had slightly differing baseline characteristics and sample sizes, but it was unclear whether these studies were using the same study sample. Third, there was heterogeneity in the reported scores for the functional measures. In some instances, overall scores were imputed, some studies did not allow for score imputation, and some authors reported raw scores or z-scores that could not be used for assessing correlations between the UPSA and the functional measures. Fourth, living situation and employment status were presented idiosyncratically across studies; therefore, comparability of these results was facilitated by creating standardized categories. Fifth, aggregated data was summarized across studies, which is prone to ecological bias. Metaregression could have been used to adjust functional estimates for differences in study characteristics, but there were insufficient data to conduct such analyses. Sixth, changes in the correlation of the UPSA with functional measures overtime could not be assessed, as most studies were cross-sectional (at least with respect to measuring functional status), and the few that were longitudinal did not evaluate measures of interest. Finally, conclusions about which functional measures correlate best with the UPSA are based on the amount of evidence available to investigate those associations at present; further research is required to fully understand the value of functional measures with little published data.

## 6. Conclusion

This review provides data on the association between functional capacity, as measured by the UPSA, and other functional measures in schizophrenia, and the strength of those associations. Of all the functional measures considered, the amount of evidence was greatest for the relationship between the UPSA and the SLOF, and the SLOF has the strongest observed correlation with the UPSA. Authors of studies that evaluated the GAF and the QLS found that these measures correlated well with the UPSA. Although evidence supports a relationship between the UPSA and functional measures that assess real-world outcomes in patients with schizophrenia, further evaluation of these relationships is needed in order to maximize their implementation in trials, as well as determine the need for and inform the subsequent development of new assessments. Regardless, the findings of this study may help inform the design of upcoming trials of schizophrenia treatments, as well as contribute to the framework for understanding the clinical and economic value of emerging treatments.

## Figures and Tables

**Table 1:** Availability of measures in the identified studies.

**Measure** ****	**Studies ** **that ** **reported ** **measure**	
	**N**	** (**%**)**
UCSD Performance-based Skills Assessment (UPSA)^a^	73	(100)
Full UPSA	41	(56)
UPSA-B	33	(45)
UPSA-2	2	(3)
UPSA-M	1	(1)
C-UPSA	1	(1)
UPSA-VIM	1	(1)
Specific Level of Function (SLOF)	25	(34)
Global Assessment of Functioning (GAF)	11	(15)
Quality of Life Scale (QLS)	7	(10)
Multidimensional Scale of Independent Functioning (MSIF)	6	(8)
Quality of Life Interview (QOLI)	5	(7)
Strauss-Carpenter Level of Function	2	(3)
Personal and Social Performance Scale (PSP)	2	(3)
Role Functioning Scale (RFS)	4	(5)
Scale of Functioning (SOF)	2	(3)
Independent Living Skills Survey (ILSS)	2	(3)
Social Functioning Scale (SFS)	3	(4)
Quality of Well-being Scale (QWB)	2	(3)
Independent Living Scale (ILS)	1	(1)
Independent Living Skills Inventory (ILSI)	1	(1)
Medical Outcomes Survey – Short-form 36 (SF-36)	1	(1)
Life Skills Profile (LSP)	1	(1)
Social Behavior Scale (SBS)	1	(1)
Residential Status - Outcome	7	10)
Employment Status - Outcome	5	(7)
Residential Status - Baseline characteristics	36	(49)
Employment Status - Baseline characteristics	19	(26)

^a^There were six studies that reported both the UPSA and the UPSA-B.

**Table 2:** Baseline characteristics of the patient populations in the identified studies.

**Study**	**N**	%** Male**	**Age at baseline ** **Mean (SD)**	%** Caucasian**	**Years of education ** **Mean (SD)**	%** with schizophrenia diagnosis**	**Years since diagnosis**	%** Use of anti-psychotics**
Gupta M et al., 2012	54	NA	38 (9.3)	NA	NA	100	NA	NA
Twamley EW et al., 2002	111	52	55 (9.6)	75	13 (2.5)	60	27	91
Mausbach BT et al., 2010	116	60	50 (9.0)	NA	14 (2.4)	100	NA	93
Abram SV et al., 2014	59	63	35 (9.4)	44	NA	100	14.8	NA
Alden EC et al., 2015	36	61	37 (9.1)	44	NA	100	15	NA
Bowie CR et al., 2008	222	NA	57 (9.7)	NA	13 (2.5)	NA	NA	100
Bowie CR et al., 2006	78	NA	58 (7.3)	NA	13 (2.4)	100	NA	100
Bowie CR et al., 2007	67	76	59 (13.7)	61	12 (1.7)	76	NA	NA
Cardenas V et al., 2013	97	57	51 (6.5)	50	12 (2.3)	75	NA	NA
Depp CA et al., 2011	73	53	50 (6.3)	45	12 (2.1)	NA	NA	89
Depp CA et al., 2016	196	62	52 (7.3)	53	12 (2.3)	NA	NA	NA
Durand D et al., 2015	214	65	41 (12.4)	55	12 (2.2)	NA	NA	NA
Galderisi S et al., 2014	921	70	40 (10.7)	NA	12 (3.4)	100	NA	97
Gold JM et al., 2012	114	55	39 (11.5)	57	13 (2.2)	NA	NA	NA
Gould F et al., 2012	194	73	59 (7.9)	NA	12 (4.4)	NA	NA	100
Harvey PD et al., 2009	146	63	48 (11.9)	100	14 (3.4)	NA	NA	100
Harvey PD et al., 2009	236	70	57 (7.3)	64	13 (2.2)	NA	NA	100
Ho JS et al., 2013	138	67	51 (6.9)	59	NA	84	NA	100
Holshausen K et al., 2014	148	72	56 (7.6)	57	13 (2.5)	NA	NA	100
Leifker FR et al., 2009	194	NA	NA	NA	NA	NA	NA	NA
Leung WW et al., 2008	230	73	56 (9.9)	60	13 (2.6)	77	28.4	NA
Olsson AK et al., 2012	211	64	49 (11.6)	NA	NA	65	NA	NA
Smith MJ et al., 2012	46	65	35 (8.2)	51	NA	100	14.5	100
Garcia-Portilla MP et al., 2013	139	73	40 (10.4)	NA	NA	100	NA	NA
Kim SJ et al., 2015	55	62	36 (6.9)	NA	13 (2.3)	100	11	100
Ucok A et al., 2012	295	45	42 (15.4)	NA	14 (4.2)	NA	NA	NA
Bengoetxea E et al., 2014	29	48	30 (9.2)	NA	9 (3.3)	NA	NA	NA
Lee J et al., 2015	351	67	47 (11)	55	12 (1.9)	NA	NA	NA
Light GA et al., 2005	25	64	39 (9.6)	NA	12 (1.7)	100	17.7	88
Light GA et al., 2012	178	72	43 (10.1)	NA	NA	NA	21.9	NA
Musso MW et al., 2014	18	89	38 (12.3)	60	NA	NA	NA	100
Vesterager L et al., 2012	117	54	25 (3.3)	NA	NA	84	NA	89
Elliott CS et al., 2014	71	75	48 (9.4)	NA	12 (1.9)	NA	NA	NA
Fiszdon JM et al., 2010	48	81	49 (8.7)	56	12 (1.8)	83	NA	NA
Twamley EW et al., 2012	69	65	46 (9.5)	59	13 (1.7)	54	23.3	94
Mausbach BT et al., 2008	236	65	49 (7.3)	53	NA	80	NA	NA
Narvaez JM et al., 2008	88	67	47 (8.8)	66	13 (2.4)	42	21.9	92
Twamley EW et al., 2011	89	65	47 (9.8)	58	13 (1.9)	51	25.7	88
Ammari N et al., 2014	72	80	42 (9.8)	NA	13 (2.0)	NA	NA	NA
Heinrichs RW et al., 2010	127	65	42 (9.0)	NA	NA	NA	NA	99
Heinrichs RW et al., 2006	64	75	40 (8.9)	NA	NA	NA	NA	95
Muharib E et al., 2014	35	80	31 (5.9)	66	13 (2.5)	NA	8.5	92
Sheffield JM et al., 2014	104	58	40 (11.9)	61	13 (3.9)	NA	NA	NA
Silverstein SM et al., 2011	155	65	0 (0)	48	NA	100	NA	NA
Green MF et al., 2011	163	66	44 (10.1)	NA	12 (2.1)	100	20.3	NA
Roseman AS et al., 2008	144	80	52 (8.6)	55	12 (2.2)	65	NA	NA
Adelsky MB et al., 2011	50	48	58 (8.7)	40	11 (2.7)	100	NA	NA
Green MF et al., 2008	176	76	44 (11.2)	59	12 (2.4)	86	NA	97
Helldin L et al., 2012	95	44	36 (8.2)	NA	11 (2.5)	NA	NA	NA
Jeste ND et al., 2005	136	59	49 (7.5)	NA	11 (2.3)	81	23.0	NA
Kasckow J et al., 2007	145	78	52 (145.0)	56	12 (2.3)	62	NA	NA
Keefe RS et al., 2006	56	84	35 (9.8)	NA	12 (2.1)	100	NA	100
Mausbach BT et al., 2008	434	66	50 (7.9)	67	12 (2.4)	77	NA	NA
Mausbach BT et al., 2011	367	63	51 (9.6)	NA	NA	NA	NA	NA
McClure MM et al., 2013	46	61	38 (11.8)	NA	NA	0	NA	NA
Moore RC et al., 2015	21	55	51 (8.4)	45	13 (1.9)	NA	NA	NA
Moore RC et al., 2013	21	76	55 (4.1)	76	13 (2.1)	NA	NA	NA
Murthy NV et al., 2012	55∗	76	31 (7.0)	NA	12 (2.1)	100	6.4	NA
Patterson TL et al., 2001	50	42	56 (8.5)	74	13 (2.3)	NA	29.4	NA
Stergiopoulos V et al., 2011	51	71	40 (11.6)	65	12 (2.6)	70	NA	71
Thorp SR et al., 2012	746	NA	56 (7.8)	68	12 (2.8)	NA	NA	NA
Twamley EW et al., 2012	30	54	52 (5.1)	57	12 (2.7)	32	23.4	89
Vahia IV et al., 2010	884	64	52 (8.3)	64	12 (2.5)	NA	24.9	NA
Strassnig MT et al., 2015	402	66	50 (5.1)	57	12 (5.1)	100	NA	NA
Sabbag S et al., 2011	193	69	44 (11.5)	54	13 (2.4)	100	NA	NA
Bechi M et al., 2017	79	62	41 (10.3)	NA	NA	100	NA	100
Czaja SJ et al., 2017	77	73	54 (7.6)	26	NA	100	NA	NA
Keefe RSE et al., 2016	158	56	43 (11.9)	47	13 (2.0)	100	NA	NA
Koshikawa Y et al., 2016	21	52	45 (11.1)	NA	NA	95	NA	NA
Lee J et al., 2017	60	53	64 (6.8)	NA	12 (3.1)	NA	NA	NA
Moore RC et al., 2015^a^	34	55	44 (9.2)	NA	12 (2.4)	NA	NA	100
Moore RC et al., 2015^b^	435	64	50 (10.0)	NA	14 (2.0)	NA	NA	NA
Ventura J et al., 2016	205	61	52 (7.0)	54	12 (2.0)	NA	NA	NA
Kumar S et al., 2016	98	70	25 (5.1)	45	13 (1.8)	69	NA	NA

NA, not applicable; SD, standard difference.

^a^Patients in Study 1. ^b^Patients in Study 2.

## Supplementary Materials

Supplementary Materials Supplementary Table 1: criteria to define the scope of the systematic literature review. Supplementary Table 2: description of and references for scales to measure function among patients with schizophrenia. Supplementary Table 3: design characteristics of the studies included in the systematic review. Supplementary Table 4: mean (SD) scores on, and correlations between, the UPSA and the SLOF. Supplementary Table 5: mean (SD) scores on, and correlations between, the UPSA and the GAF. Supplementary Table 6: mean (SD) scores on, and correlations between, the UPSA and MSIF. Supplementary Table 7: study samples according to baseline residential status and mean (SD) UPSA score. Supplementary Table 8: study samples according to baseline employment status and mean (SD) UPSA score. Supplementary Table 9: STROBE statement checklist for included studies.
