# Supplementary material for: Assessing the Relationship between Performance on the University of California Performance Skills Assessment (UPSA) and Outcomes in Schizophrenia: A Systematic Review and Evidence Synthesis
Source: Schizophr Res Treatment. 2018 Dec 27;2018:9075174. doi: 10.1155/2018/9075174 (PMC6327277; doi:10.1155/2018/9075174)
Supplement: Supplementary Materials — Supplementary Table 1: criteria to define the scope of the systematic literature review. Supplementary Table 2: description of and references for scales to measure function among patients with schizophrenia. Supplementary Table 3: design characteristics of the studies included in the systematic review. Supplementary Table 4: mean (SD) scores on, and correlations between, the UPSA and the SLOF. Supplementary Table 5: mean (SD) scores on, and correlations between, the UPSA and the GAF. Supplementary Table 6: mean (SD) scores on, and correlations between, the UPSA and MSIF. Supplementary Table 7: study samples according to baseline residential status and mean (SD) UPSA score. Supplementary Table 8: study samples according to baseline employment status and mean (SD) UPSA score. Supplementary Table 9: STROBE statement checklist for included studies. [file 9075174.f1.pdf]

Supplementary Table 1: Scope of the literature review

**Interventions/ comparators**	NA
**Outcomes**	Estimates of the frequency of key functional outcomes, including:<br>• Residential status<br>• Employment status<br>• Generic or disease-specific HRQoL scores<br>• Scores on other functional scale measures
**Study design**	• Prospective cohort studies / registries<br>• Retrospective cohort studies / database studies<br>• Randomized controlled trials

HRQoL, health-related quality of life; NA, not applicable; UPSA, UCSD Performance-based Skills Assessment

Supplementary Table 2: Description of scales

Scale	Brief description	Reference
UCSD Performance-based Skills Assessment (UPSA)	A performance based measure of functional skill competence using role play scenarios. The five domains include: (1) household chores, (2) communication, (3) finance, (4) transportation, and (5) planning recreational activities.	Patterson, 2001
Specific Level of Function (SLOF)	An interview based instrument that measures directly observable behavioural functioning- focusing on an individual's skills, assets, and abilities. The scale is administered to a key informant (caseworker, caregiver, family member, friend). Six domains include (1) physical functioning, (2) personal care skills, (3) interpersonal relationships, (4) social acceptability, (5) activities of community living, (6) work skills.	Schneider, 1983

Global Assessment of Functioning (GAF)	A clinician rated measure of overall impairment caused by mental factors. Can be used to communicate the level of impairment, indicate the need of professional help, and reflect improvement or change over time. Assesses global function in terms of three domains: (1) psychological, (2) social and (3) occupational functioning.	Jones, 1995
Quality of Life Scale (QLS)	A semi structured patient interview, clinician rated instrument to assess function over the past four weeks. Four domains include 1) Intrapsychic foundations (2) interpersonal relations (3) instrumental role, (4) common objects & activities.	Heinrichs, 1984
Multidimensional Scale of Independent Functioning (MSIF)	A semi structured patient interview, clinician rated scale which captures independent functioning over a one month time period. Ratings are made using a set of detailed rating anchors. Three domains are captured: (1) work, (2) education, and (3) residence.	Jaeger, 2003
Quality of Life Interview (QOLI)	A structured self-report patient interview, administered by a trained interviewer. Global life satisfaction and objective and subjective quality of life are measured in eight domains: (1) living situation, (2) daily activities and functioning, (3) family relations, (4) social relations, (5) finances, (6) work and school, (7) legal and safety issues, and (8) health.	Lehman, 1988
Strauss-Carpenter Level of Function	A semi structured interviewer administered scale including four domains: (1) social contacts (frequency and quality of social contacts), (2) work (quantity and quality of useful work), (3) symptomatology (absence of symptoms or recent hospitalizations) and (4) function (fullness of life and overall level of function).	Strauss, 1977

Personal and Social Performance Scale (PSP)	A clinician rated scale designed to measure and distinguish between specific domains of functioning. The four domains measured are: (1) socially useful activities, (2) personal and social relationships, (3) self-care, (4) disturbing and aggressive behaviours.	Morosini, 2000
Role Functioning Scale (RFS)	An interview administered and rated scale which evaluates the functioning of individuals in areas of everyday life. The four domains include (1) working and productivity, (2) independent living and self care, (3) immediate social network relationships, and (4) extended social network relationships.	Goodman, 1993
Scale of Functioning (SOF)	A trained interviewer administered scale to assess functioning in individuals with schizophrenia.	Rapaport, 1996
Independent Living Skills Survey (ILSS)	A survey to assess functioning over the past 30 days. Two versions exist: informant and self-report. Self-report version is appropriate when individual does not have anyone who is sufficiently knowledgeable of their daily functioning and is in questionnaire format. Twelve areas of basic community living are assessed.	Wallace, 2000
Social Functioning Scale (SFS)	An informant interview developed to assess social adjustment in individuals with schizophrenia. Assesses seven domains: (1) social engagement/withdrawal, (2) interpersonal behaviour/communication (3) prosocial activities (4) recreation (5) independence-competence (6) independence-performance and (7) employment/occupation.	Birchwood, 1990
Quality of Well-being Scale (QWB)	A preference-weighted measure of symptoms and functioning. The three domains include (1) mobility, (2) physical activity, and (3) social activity.	Kaplan, 1989

Independent Living Scale (ILS)	An interviewer administered scale to assess the likelihood of successful independent living. The five domains include (1) memory-orientation, (2) managing money, (3) managing home and transportation, (4) health and safety", and (5) social adjustment.	Revheim, 2004
Independent Living Skills Inventory (ILSI)	A multifaceted, functional assessment that measures an individual's ability over the previous 30 days. The eleven subscales include hygiene and grooming, (3) clothing, (4) basic skills, (5) interpersonal skills, (6) home maintenance, (7) money management, (8) cooking, (9) resource utilization, (10) general occupational skills, and (11) medication management.	Menditto, 1999
Medical Outcomes Survey – Short-form 36 (SF-36)	A measure that assesses general health concepts relevant across age, disease, and treatment groups. Eight domains include (1) physical functioning, (2) role physical, (3) bodily pain, (4) general health, (5) vitality, (6) social functioning (7) role-emotional, and (8) mental health.	Ware, 1992
Life Skills Profile (LSP)	An informant survey that measures an individuals' functional status over the previous three months. Five domains include (1) self-care, (2) nonturbulence, (3) social contact, (4) communication, and (5) responsibility.	Rosen, 1989
Social Behavior Scale (SBS)	A semi structured interview delivered to a key informant, asked to describe an individuals' functioning over the past month. A total of 21 functional areas are assessed.	Wykes, 1986

References:
- Patterson TL, Goldman S, McKibbin CL, Hughs T, Jeste DV. UCSD Performance-Based Skills Assessment: Development of a New Measure of Everyday Functioning for Severely Mentally Ill Adults. Schizophrenia Bulletin. 2001;27(2):235-245.
- Schneider LC, Struening EL. SLOF: a behavioral rating scale for assessing the mentally ill. Paper presented at: Social Work Research and Abstracts 1983.
- Jones SH, Thornicroft G, Coffey M, Dunn G. A brief mental health outcome scale-reliability and validity of the Global Assessment of Functioning (GAF). The British Journal of Psychiatry. 1995;166(5):654-659.
- Heinrichs DW, Hanlon TE, Carpenter Jr WT. The Quality of Life Scale: an instrument for rating the schizophrenic deficit syndrome. Schizophrenia bulletin. 1984;10(3):388-398.
- Jaeger J, Berns SM, Czobor P. The multidimensional scale of independent functioning: a new instrument for measuring functional disability in psychiatric populations. Schizophrenia bulletin. 2003;29(1):153-167.
- Lehman AF. A quality of life interview for the chronically mentally ill. Evaluation and program planning.1988;11(1):51-62.

- Strauss JS, Carpenter WT. Prediction of outcome in Schizophrenia: III. Five-yearn outcome and its predictors. Archives of General Psychiatry. 1977;34(2):159-163.
- Morosini P, Magliano L, Brambilla L, Ugolini S, Pioli R. Development, reliability and acceptability of a new version of the DSM-IV Social and Occupational Functioning Assessment Scale (SOFAS) to assess routine social funtioning. Acta Psychiatrica Scandinavica. 2000;101(4):323-329.
- Goodman SH, Sewell DR, Cooley EL, Leavitt N. Assessing levels of adaptive functioning: the Role Functioning Scale. Community mental health journal. 1993;29(2):119-131.
- Rapaport MH, Bazzetta J, McAdams LA, Patterson T, Jeste DV. Validation of the scale of functioning in older outpatients with schizophrenia. The American Journal of Geriatric Psychiatry. 1996;4(3):218-228.
- Wallace CJ, Liberman RP, Tauber R, Wallace J. The Independent Living Skills Survey: A comprehensive measure of the community functioning of severely and persistently mentally ill individuals. Schizophrenia bulletin. 2000;26(3):631.
- Birchwood M, Smith J, Cochrane R, Wetton S, Copestake S. The Social Functioning Scale. The development and validation of a new scale of social adjustment for use in family intervention programmes with schizophrenic patients. The British Journal of Psychiatry. 1990;157(6):853-859.
- Kaplan RM, Anderson JP, Wu AW, Mathews WC, Kozin F, Orenstein D. The Quality of Well-being Scale: applications in AIDS, cystic fibrosis, and arthritis. Medical care. 1989:S27-S43.
- Revheim N, Medalia A. The independent living scales as a measure of functional outcome for schizophrenia. Psychiatric Services. 2004;55(9):1052-1054.
- Menditto AA, Wallace CJ, Liberman RP, Wal JV, Jones NT, Stuve P. Functional assessment of independent living skills. Psychiatric Rehabilitation Skills. 1999;3(2):200-219.
- Ware Jr JE, Sherbourne CD. The MOS 36-item short-form health survey (SF-36): I. Conceptual framework and item selection. Medical care. 1992:473-483.
- Rosen A, Hadzi-Pavlovic D, Parker G. The life skills profile: a measure assessing function and disability in schizophrenia. Schizophrenia Bulletin. 1989;15(2):325-337.
- Wykes T, Sturt E. The measurement of social behaviour in psychiatric patients: an assessment of the reliability and validity of the SBS schedule. The British Journal of Psychiatry. 1986;148(1):1-11.

Supplementary Table 3: Characteristics of the included studies

Study	Title	Study objective	Study design	Sample type	Measures reported	Intervention details
Gupta M et al., 2012	Predictors of change in functional competence and functional behavior after functional	To identify baseline predictors of the acquisition of functional competence and real-world performance in adaptive and interpersonal domains	Prospective cohort selected from an RCT	Community-dwelling outpatients	UPSA; SLOF	All patients received an intervention to help improve independent living skills

Study	Title	Study objective	Study design	Sample type	Measures reported	Intervention details
	adaptation skills training for schizophrenia.	during psychosocial intervention.				
Twamley EW et al., 2002	Generalized cognitive impairments, ability to perform everyday tasks, and level of independence in community living situations of older patients with psychosis.	To examine the relationship between performance of instrumental activities of daily living, as measured with the University of California at San Diego Performance-Based Skills Assessment (UPSA), and measures of cognitive functioning and independence in the community living situation of older outpatients with psychotic disorders.	Cross-sectional	Mixed community-dwelling and institutionalized patients; from university and clinic-based sites, as well as the community	UPSA; residential status	NA

Study	Title	Study objective	Study design	Sample type	Measures reported	Intervention details
Mausbach BT et al., 2010	Relationship of the Brief UCSD Performance-based Skills Assessment (UPSA-B) to multiple indicators of functioning in people with schizophrenia and bipolar disorder.	To assess the relationship between multiple indicators of 'real-world' functioning and scores on a brief performance-based measure of functional capacity known as the Brief University of California at San Diego (UCSD) Performance-based Skills Assessment (UPSA- B) in a sample of 205 patients with either serious bipolar disorder (n = 89) or schizophrenia (n = 116).	Cross-sectional	Mixed community-dwelling and residential treatment setting patients	UPSA-B; residential status; SLOF; employment status	NA
Abram SV et al., 2014	Accurate perception of negative emotions predicts functional capacity in schizophrenia.	The present study examined whether impairment in the perception of specific emotional valences (positive, negative) and neutrality were uniquely associated with social functioning, using a multimodal social functioning battery.	Cross-sectional	Community-dwelling outpatients	UPSA-B; SLOF	NA
Alden EC et al., 2015	Cluster analysis differentiates high and low community functioning in schizophrenia:	We characterized schizophrenia subjects based on their level of community functioning through cluster analysis in an effort to identify whether	Cross-sectional	Community-dwelling outpatients	UPSA-B; SLOF	NA

Study	Title	Study objective	Study design	Sample type	Measures reported	Intervention details
	subgroups differ on working memory but not other neurocognitive domains.	specific neurocognitive domains were associated with variation in functioning.				
Bowie CR et al., 2008	Predicting schizophrenia patients' real-world behavior with specific neuropsychological and functional capacity measures.	To examine the different predictive relationships between NP domains, functional competence, social competence, symptoms, and real-world behavior in domains of work skills, interpersonal relationships, and community activities.	Cross-sectional	Community-dwelling outpatients	UPSA; SLOF	NA
Bowie CR et al., 2006	Determinants of real-world functional performance in schizophrenia subjects: correlations with cognition, functional capacity, and symptoms.	Our analyses were aimed at determining if 1) neuropsychological performance would be associated with real-world outcome other than through its relationship with functional capacity, and 2) whether other factors, such as depression or negative symptoms, would affect outcome and, if so, were these influences at the level of affecting functional capacity and	Cross-sectional	Community-dwelling outpatients	UPSA; SLOF	NA

Study	Title	Study objective	Study design	Sample type	Measures reported	Intervention details
		neuropsychological performance or real-world performance.				
Bowie CR et al., 2007	Self-assessment of functional status in schizophrenia.	The purpose of this study was to examine the convergence of schizophrenia patients' reports of their everyday functional status (using a self-report of real-world functional outcomes) with the reports of their case managers, and to identify the correlates of the level of accuracy of these reports.	Cross-sectional	Community-dwelling outpatients	UPSA; SLOF	NA
Cardenas V et al., 2013	When functional capacity and real-world functioning converge: the role of self-efficacy.	To test the hypothesis that the relationship between functional capacity and real-world functioning, as measured by both proxy and self-report, would be moderated by the individual's self-efficacy, such that functional capacity would be more strongly correlated with real-world functioning when self-efficacy was high versus low.	Cross-sectional	Community-dwelling outpatients	UPSA-B; SLOF	NA

Study	Title	Study objective	Study design	Sample type	Measures reported	Intervention details
Depp CA et al., 2011	Psychopathological and functional correlates of behavioral activation and avoidance in schizophrenia.	The goals of our study were to examine the associations between BADS subscales and positive and negative symptoms of schizophrenia and depressive symptoms, and to examine the associations between activation and avoidance with measures of cognitive ability, functional capacity, observer-rated functioning, and perceived functioning.	Cross-sectional	Community-dwelling outpatients	UPSA; SLOF	NA
Depp CA et al., 2016	Neurocognitive and functional correlates of mobile phone use in middle-aged and older patients with schizophrenia.	To examine the association of mobile phone use and ownership with psychopathology, cognitive functioning, and functional outcome in 196 outpatients aged 40 years and older who were diagnosed with schizophrenia.	Cross-sectional	Community-dwelling; independent or semi-independent (for example, apartment supervised by treatment program)	UPSA-B; SLOF	NA
Durand D et al., 2015	Factors influencing self-assessment of cognition and functioning in schizophrenia: implications for treatment studies.	To present a systematic study of the validity of self-reported versus clinician-reported cognitive performance.	Cross-sectional	Community-dwelling outpatients	UPSA-B; SLOF	NA

Study	Title	Study objective	Study design	Sample type	Measures reported	Intervention details
Galderisi S et al., 2014	The influence of illness-related variables, personal resources and context-related factors on real-life functioning of people with schizophrenia.	The goal of the present study was to identify predictors of real-life functioning in people with schizophrenia, and to assess their relative contribution.	Cross-sectional	Community-dwelling outpatients	UPSA-B; SLOF	NA
Gold JM et al., 2012	Clinical, functional, and intertask correlations of measures developed by the Cognitive Neuroscience Test Reliability and Clinical Applications for Schizophrenia Consortium.	The goal of the Cognitive Neuroscience Test Reliability and Clinical Applications for Schizophrenia (CNTRACS) Consortium was to develop measures of discrete cognitive processes, allowing for the interpretation of specific deficits that could be linked to specific neural systems.	Cross-sectional	Community-dwelling outpatients OR partial hospital status	UPSA-B; SLOF	NA
Gould F et al., 2012	The influence of demographic factors on functional capacity and everyday functional	We examined the correlation of demographic factors (race, age, and education) and FC measures, and the relative ability of NP performance, FC, and demographic factors to predict real-world	Cross-sectional	Community-dwelling outpatients	UPSA-B; SLOF	NA

Study	Title	Study objective	Study design	Sample type	Measures reported	Intervention details
	outcomes in schizophrenia.	outcomes in social, vocational, and residential domains in 194 outpatients with schizophrenia.				
Harvey PD et al., 2009	Performance-based measurement of functional disability in schizophrenia: a cross-national study in the United States and Sweden.	This study examined the similarity of performance-based assessments of everyday functioning, real-world disability, and achievement of milestones in people with schizophrenia in the United States and in Sweden.	Cross-sectional	Community-dwelling outpatients	UPSA-B; SLOF	NA
Harvey PD et al., 2009	Abbreviated neuropsychological assessment in schizophrenia: prediction of different aspects of outcome.	The aim of this study was to identify the best subset of neuropsychological tests for prediction of several different aspects of functioning in a large (n = 236) sample of older people with schizophrenia.	Cross-sectional	Community-dwelling outpatients	UPSA-B; SLOF	NA
Ho JS et al., 2013	Direct and mediated effects of cognitive function with multidimensional outcome	We examined the extent to which functional capacity (i.e., skills necessary to live independently) mediated the relationship between cognitive ability and both	Cross-sectional	Community-dwelling outpatients	UPSA; SLOF	NA

Study	Title	Study objective	Study design	Sample type	Measures reported	Intervention details
	measures in schizophrenia: the role of functional capacity.	observer and self-reported, real-world functioning in 138 outpatients with schizophrenia.				
Holshausen K et al., 2014	Neurocognition, functional capacity, and functional outcomes: the cost of inexperience.	In this study, we evaluated the impact of prior experience with performing functionally skilled acts on real-world functional outcomes. We hypothesized that experience in the performance of skilled acts would significantly predict scores on performance-based assessments of functional capacity but that neurocognition would still emerge as the strongest predictor of impairments in functional capacity. Further, we hypothesized that prior experience and functional capacity would be significant predictors of real-world functioning, regardless of other influences.	Cross-sectional	Community-dwelling outpatients	UPSA; SLOF	NA

Study	Title	Study objective	Study design	Sample type	Measures reported	Intervention details
Leifker FR et al., 2009	Determinants of everyday outcomes in schizophrenia: the influences of cognitive impairment, functional capacity, and symptoms.	This study examined the relative importance of cognitive impairments measured with a neuropsychological battery, performance-based measures of social and everyday living skills, and positive and negative symptoms for the prediction of real-world outcomes in social and residential domains.	Cross-sectional	Community-dwelling, including nursing homes	UPSA-B; SLOF	NA
Leung WW et al., 2008	Functional implications of neuropsychological normality and symptom remission in older outpatients diagnosed with schizophrenia: a cross-sectional study.	The goal of this investigation was to examine whether neuropsychological and cross-sectional symptomatic status differentially predict elements of functional outcome.	Cross-sectional	Community-dwelling outpatients, including nursing home	UPSA; SLOF	NA
Olsson AK et al., 2012	Psychometric properties of a performance-based measurement of functional capacity, the	The aim of the present study was to further explore the psychometric properties of the UPSA-B, as well as to ensure that the Swedish version can be used in	Cross-sectional	Community-dwelling outpatients	UPSA-B; GAF; Strauss-Carpenter Scale	NA

Study	Title	Study objective	Study design	Sample type	Measures reported	Intervention details
	UCSD Performance-based Skills Assessment - Brief version.	clinical practice and for research purposes.				
Smith MJ et al., 2012	Self-reported empathy deficits are uniquely associated with poor functioning in schizophrenia.	The current study examined whether impairments in self-reported empathy were associated with poor functioning, above and beyond the influences of neurocognitive deficits and psychopathology.	Cross-sectional	Community-dwelling outpatients	UPSA-B; SLOF	NA
Garcia-Portilla MP et al., 2013	Validation of a European Spanish-version of the University of California performance Skills Assessment (Sp-UPSA) in patients with schizophrenia and bipolar disorder.	To validate the Spanish version of the University of California Performance Skills Assessment (UPSA) in patients with severe mental disorders.	Cross-sectional analysis of a prospective cohort	Community-dwelling outpatients	UPSA; GAF; PSP	NA
Kim SJ et al., 2015	Differences in cognitive function and daily living skills between early-	This study was performed to assess the difference in cognitive function and daily living skills between the	Cross-sectional	Community-dwelling outpatients	UPSA	NA

Study	Title	Study objective	Study design	Sample type	Measures reported	Intervention details
	and late-stage schizophrenia.	early- and late-stage schizophrenia.				
Ucok A et al., 2012	Employment and its relationship with functionality and quality of life in patients with schizophrenia: EGOFORS Study.	The aim of this article is to examine the employment status of schizophrenia patients, the differences between patients who are employed and unemployed, and predictors of employment status in a large sample consisting of chronic patients. Our hypothesis was that the patients who are employed will have fewer negative symptoms and better functioning compared to the ones who are unemployed.	Cross-sectional	Community-dwelling outpatients	UPSA-B; QLS; GAF; PSP	NA
Bengoetxea E et al., 2014	The effect of language on functional capacity assessment in middle-aged and older US Latinos with schizophrenia.	This study examined the effect of language on the assessment of functional capacity in middle-aged and older Latino patients with schizophrenia.	Cross-sectional	NA	UPSA; GAF	NA
Lee J et al., 2015	Verbal working memory in schizophrenia	Using the Consortium on the Genetics of Schizophrenia case-control	Cross-sectional	NA	UPSA-B; GAF; RFS; SOF	NA

Study	Title	Study objective	Study design	Sample type	Measures reported	Intervention details
	from the Consortium on the Genetics of Schizophrenia (COGS) study: the moderating role of smoking status and antipsychotic medications.	study (COGS-2), we examined smoking status, types of antipsychotic medication, and history of substance as moderators for working memory impairment in schizophrenia.				
Light GA et al., 2005	Mismatch negativity deficits are associated with poor functioning in schizophrenia patients.	To determine if a deficit in the processing of deviant versus frequent auditory stimuli is associated with indexes of functioning and symptom severity in a heterogeneous sample of schizophrenia patients.	Cross-sectional	Community-dwelling, some from board-and-care homes	UPSA; residential status; GAF; QOLI	NA
Light GA et al., 2012	Characterization of neurophysiologic and neurocognitive biomarkers for use in genomic and clinical outcome studies of schizophrenia.	The objectives of this study were to characterize the extent to which widely-used neurophysiological and neurocognitive endophenotypes are: 1) associated with schizophrenia, 2) stable over time, independent of state-related changes, and 3) free of potential practice/maturation or differential attrition effects	Prospective cohort	Schizophrenia patients were recruited from "community residential facilities" and via physician referral. Therefore, community-dwelling but not completely independent	UPSA; GAF; SOF	NA

Study	Title	Study objective	Study design	Sample type	Measures reported	Intervention details
		in schizophrenia patients (SZ) and nonpsychiatric comparison subjects (NCS). Stability of clinical and functional measures was also assessed.				
Musso MW et al., 2014	Investigation of the Montreal Cognitive Assessment (MoCA) as a cognitive screener in severe mental illness.	To examine the utility and psychometric properties of the MoCA in a group of outpatients with severe mental illness.	Cross-sectional	Outpatients	UPSA-2; GAF	NA
Vesterager L et al., 2012	Cognitive and clinical predictors of functional capacity in patients with first episode schizophrenia.	To investigate predictors of functional capacity in first episode schizophrenia and the associations between functional capacity and measures of real-world functioning.	Prospective cohort selected from an RCT	Community-dwelling outpatients	UPSA-B; GAF; RFS	Patients were randomized to cognitive remediation plus a comprehensive psychosocial program or the comprehensive psychosocial program alone.
Elliott CS et al., 2014	Comparison of self-report and performance-based measures of everyday functioning in	To examine the relationship between a self-report and two performance-based measures of everyday functioning.	Cross-sectional	Community-dwelling outpatients	UPSA; ILSS	NA

Study	Title	Study objective	Study design	Sample type	Measures reported	Intervention details
	individuals with schizophrenia: implications for measure selection.					
Fiszdon JM et al., 2010	Functional significance of preserved affect recognition in schizophrenia.	In the current study, we sought to evaluate the functional significance of affect recognition (AR) deficits in schizophrenia by comparing subgroups with normal-range and impaired AR performance on proxy and interviewer-rated measures of real-world functioning.	Cross-sectional	Community-dwelling outpatients	UPSA; QLS; GAF; ILSS	NA
Twamley EW et al., 2012	Compensatory cognitive training for psychosis: effects in a randomized controlled trial.	To test the efficacy of a 12-week, manualized Compensatory Cognitive Training (CCT) intervention designed to target four cognitive domains: 1) prospective memory; 2) attention and vigilance; 3) learning and memory; and 4) executive functioning.	Randomized trial	Community-dwelling outpatients	UPSA; QOLI	Compensatory Cognitive Training (CCT) intervention
Mausbach BT et al., 2008	Relationship between functional capacity and community	The purpose of this study was threefold. First, we were interested in determining whether patients residing in	Cross-sectional	Community-dwelling outpatients (residing alone or with family) and	UPSA-B; QOLI	FAST program

Study	Title	Study objective	Study design	Sample type	Measures reported	Intervention details
	responsibility in patients with schizophrenia: differences between independent and assisted living settings.	residential facilities (e.g., board-and-care facilities, group homes, assisted living) engaged in fewer community responsibilities relative to those residing in the community (independently or with family). Our second aim was to examine potential reasons why participants residing in residential care facilities differed from those residing in the community on their level of community responsibility (e.g., older age, greater functional impairment, and greater symptoms of psychosis). Finally, we also examined whether level of community responsibility was significantly associated with scores on our brief measure of functional capacity (i.e., UPSA-B).		outpatients living in residential facilities (group homes, board-and-care)		
Narvaez JM et al., 2008	Subjective and objective quality of life in schizophrenia.	To examine the clinical, functional, and cognitive predictors of subjective and objective QOL in	Cross-sectional	Community-dwelling outpatients (baseline measures from a	UPSA; QOLI	Psychosocial treatment

Study	Title	Study objective	Study design	Sample type	Measures reported	Intervention details
		outpatients with schizophrenia.		psychosocial treatment study)		
Twamley EW et al., 2011	Compensatory cognitive training for psychosis: who benefits? Who stays in treatment?	The purpose of this study was to identify who is likely to drop out of this type of study, as well as this type of intervention, and to explore individual factors predicting treatment outcome.	Randomized trial	Community-dwelling outpatients	UPSA; QOLI	"Compensatory Cognitive Training". Following baseline assessment, participants were randomly assigned in blocks of 5 to receive CT plus standard pharmacotherapy (CT) or standard pharmacotherapy alone (SP).
Ammari N et al., 2014	Preserved, deteriorated, and premorbidly impaired patterns of intellectual ability in schizophrenia.	The main purpose of this investigation was to identify patterns of intellectual performance in schizophrenia patients suggesting preserved, deteriorated, and premorbidly impaired ability, and to determine clinical, cognitive, and	Cross-sectional	Recruited from outpatient settings	UPSA; MSIF	NA

Study	Title	Study objective	Study design	Sample type	Measures reported	Intervention details
		functional correlates of these patterns.				
Heinrichs RW et al., 2010	Cognitive performance and functional competence as predictors of community independence in schizophrenia.	The aim of this study was to assess the specific contribution of functional competence in predicting a key aspect of real-world outcome in schizophrenia: community independence.	Prospective cohort	Community outpatient	UPSA; MSIF	NA
Heinrichs RW et al., 2006	The University of California Performance Skills Assessment (UPSA) in schizophrenia.	This study evaluated the University of California Performance-based Skills Assessment (UPSA) in a Canadian outpatient schizophrenia setting.	Cross-sectional	Community-dwelling outpatients	UPSA; MSIF	NA
Muharib E et al., 2014	Community outcome in cognitively normal schizophrenia patients.	Therefore, the present study was designed to answer the following questions: (1) Does normal cognition, as defined by the MCCB, exist in the schizophrenia population? (2) If so, what magnitude of benefit do patients with average	Cross-sectional	Community-dwelling outpatients	UPSA; MSIF	NA

Study	Title	Study objective	Study design	Sample type	Measures reported	Intervention details
		cognitive ability demonstrate on measures of community independence relative to patients with impaired cognition?				
Sheffield JM et al., 2014	Common and specific cognitive deficits in schizophrenia: relationships to function.	The goals of the present study were to assess the interrelationships among tasks from the MATRICS and CNTRACS batteries, to determine the degree to which tasks from each battery capture unique variance in cognitive dysfunction in schizophrenia, and to determine the ability of tasks from each battery to predict functional outcome.	Cross-sectional	Stable outpatient or partial hospital status	UPSA-B; SLOF; MSIF	NA
Silverstein SM et al., 2011	Cognition-UPSA score relationships: a further analysis of Silverstein et al. (2010) data and some caveats.	A further analysis of previous data examining relationship between UPSA scores and cognition.	Prospective cohort	Stable outpatient, non-hospitalized	UPSA; MSIF	NA
Green MF et al., 2011	Evaluation of functionally meaningful	The authors examined the reliability, validity, and	Prospective cohort	Outpatients	UPSA; UPSA-B; QLS; ILS	NA

Study	Title	Study objective	Study design	Sample type	Measures reported	Intervention details
	measures for clinical trials of cognition enhancement in schizophrenia.	practicality of functionally meaningful measures.				
Roseman AS et al., 2008	Insight, quality of life, and functional capacity in middle-aged and older adults with schizophrenia.	To investigate the role of insight as a moderator between symptom severity and subjective QOL.	Cross-sectional	Community outpatient, including board-and-care facilities, independent/assisted living facilities, and general community	UPSA; QLS	SSRI augmentation in middle aged and older individuals with schizophrenia
Adelsky MB et al., 2011	Adaptive competence impairment and cognitive deficits in acutely ill schizophrenia patients residing in nursing homes.	The objective of this article is to determine whether many of these non-geriatric patients are placed in nursing homes because of chronicity of illness and severity of impairment, or because of the limited alternative viable housing options.	Cross-sectional	Nursing home residents with schizophrenia	UPSA-B; residential status	NA
Green MF et al., 2008	Functional co-primary measures for clinical trials in schizophrenia: results from the MATRICS Psychometric and	The goal of the current study was to describe steps to evaluate four potential co-primary measures for psychometric properties and validity.	Prospective cohort	Mostly community-dwelling but at one site participants lived in a residential	UPSA; GAF; SFS	NA

Study	Title	Study objective	Study design	Sample type	Measures reported	Intervention details
	Standardization Study.			rehabilitation facility		
Helldin L et al., 2012	A functional comparison of patients with schizophrenia between the North and South of Europe.	The main objective of this study was to compare clinical and functional outcomes of patients with schizophrenia in Italy and Sweden, with a special focus on daily functioning performance and real life milestones.	Cross-sectional	Community-dwelling, including sheltered housing	UPSA-B; Strauss-Carpenter Scale	NA
Jeste ND et al., 2005	Predictors of everyday functioning among older Mexican Americans vs. Anglo-Americans with schizophrenia.	To assess the clinical, demographic and cognitive predictors of everyday functioning in Mexican Americans and Anglo American outpatients with schizophrenia.	Cross-sectional analysis of an RCT	Community-dwelling outpatients, ranged from living alone to assisted living	UPSA; residential status	FAST and PEDAL programs
Kasckow J et al., 2007	Suicidality in middle aged and older patients with schizophrenia and depressive symptoms: relationship to functioning and quality of life.	To examine the relationship between suicidality in patients with schizophrenia and quality of life, everyday functioning, social skills, and medical management.	Cross-sectional analysis of an RCT	Community-dwelling outpatients	UPSA; QLS	NA

Study	Title	Study objective	Study design	Sample type	Measures reported	Intervention details
Keefe RS et al., 2006	The relationship of the Brief Assessment of Cognition in Schizophrenia (BACS) to functional capacity and real-world functional outcome.	The purpose of this study was to determine the relationship between the BACS and a potential co-primary measure for treatment studies of cognition in schizophrenia, and to determine if such a measure accounts for significant variance in functioning beyond that provided by cognitive function.	Cross-sectional	Hospital inpatients at a rehabilitation centre	UPSA; ILSI	NA
Mausbach BT et al., 2008	Usefulness of the UCSD performance-based skills assessment (UPSA) for predicting residential independence in patients with chronic schizophrenia.	The objective of this study was to examine the sensitivity and specificity of a performance-based measure of functional capacity, the UCSD Performance-Based Skills Assessment (UPSA) for the prediction of independent living status in patients with chronic schizophrenia-related conditions	Cross-sectional	Community-dwelling outpatients (including board-and-care homes, assisted living)	UPSA; residential status	NA
Mausbach BT et al., 2011	Sensitivity and specificity of the UCSD Performance-based Skills Assessment	The purpose of this study is to examine the usefulness of the UPSA-B as a valid measure for assessing real-world functioning in a novel	Cross-sectional	Community-dwelling outpatients (including board-and-care homes, assisted living)	UPSA-B; residential status, employment status	NA

Study	Title	Study objective	Study design	Sample type	Measures reported	Intervention details
	(UPSA-B) for identifying functional milestones in schizophrenia.	sample of patients with schizophrenia and with novel outcomes (i.e., employment status).				
McClure MM et al., 2013	Functional outcomes, functional capacity, and cognitive impairment in schizotypal personality disorder.	To examine functional capacity and real-world functioning of patients with schizotypal personality disorder.	Cross-sectional	Community-dwelling outpatients	UPSA-B; residential status, employment status	NA
Moore RC et al., 2015	UPSA-M: feasibility and initial validity of a mobile application of the UCSD Performance-Based Skills Assessment.	This study aimed to develop and validate a tablet/mobile application version of the UCSD Performance-Based Skills Assessment (UPSA-M).	Cross-sectional analysis of an RCT	Community-dwelling outpatients (including board-and-care homes, assisted living)	UPSA; UPSA-B; UPSA-M; UPSA-M-Brief	Psychosocial treatments
Moore RC et al., 2013	Initial validation of a computerized version of the UCSD Performance-Based Skills Assessment (C-UPSA) for	This study aimed to validate the Computerized UCSD Performance-Based Skills Assessment (C-UPSA), a newly developed scale for assessing functional capacity in patients with schizophrenia.	Cross-sectional from an RCT - measures done at 18-month follow-up	Community-dwelling outpatients (specifically recruited from board-and-Care/assisted living facilities)	UPSA; C-UPSA; residential status	STEP program

Study	Title	Study objective	Study design	Sample type	Measures reported	Intervention details
	assessing functioning in schizophrenia.					
Murthy NV et al., 2012	Computerized cognitive remediation training for schizophrenia: an open label, multi-site, multinational methodology study.	Our study evaluated the BFP training effects in an open-label, multi-site, multinational clinical trial.	Randomized trial	Community-dwelling outpatients	UPSA-2; residential status	Brain Fitness Program (BFP)
Patterson TL et al., 2001	UCSD Performance-Based Skills Assessment: development of a new measure of everyday functioning for severely mentally ill adults.	To describe the UPSA; primary citation for this measure.	Cross-sectional	Community-dwelling outpatients	UPSA; QWB; DAFS	NA
Stergiopoulos V et al., 2011	Housing status as an independent predictor of functional capacity in patients with schizophrenia.	This study compared the functional capacity and neurocognitive status of homeless and housed adults with schizophrenia or schizoaffective disorder, and examined whether housing status is an	Cross-sectional	Homeless patients and matched patient controls from a psychiatric inpatient unit	UPSA; residential status	NA

Study	Title	Study objective	Study design	Sample type	Measures reported	Intervention details
		independent predictor of functional capacity.				
Thorp SR et al., 2012	Older patients with schizophrenia: does military veteran status matter?	The objective of this study was to examine the influence of military veteran status within a data set of older patients with schizophrenia or schizoaffective disorder.	Cross-sectional	Community-dwelling outpatients	UPSA; employment status; SF-36	NA
Twamley EW et al., 2012	The efficacy of supported employment for middle-aged and older people with schizophrenia.	The purpose of this study was to evaluate the efficacy of supported employment for middle-aged or older people with schizophrenia.	Randomized trial	Community-dwelling outpatients	UPSA; employment status	Participants were randomly assigned to receive Individual Placement and Support (IPS; the manualized version of supported employment) or conventional vocational rehabilitation (CVR) for one year
Vahia IV et al., 2010	Is late-onset schizophrenia a subtype of schizophrenia?	To determine whether late-onset schizophrenia (LOS, onset after age 40) should be	Cross-sectional	Community-dwelling outpatients including assisted living facilities	UPSA; QWB	NA

Study	Title	Study objective	Study design	Sample type	Measures reported	Intervention details
		considered a distinct subtype of schizophrenia.				
Strassnig MT et al., 2015	Determinants of different aspects of everyday outcome in schizophrenia: the roles of negative symptoms, cognition, and functional capacity.	To assess differential aspects of disability and their potential determinants. Hypothesized that negative symptoms would predict social outcomes, but not vocational functioning or everyday activities and that cognition and functional capacity would predict vocational functioning and everyday activities but not social outcomes.	Cross-sectional	Outpatient population, including community and residential care facilities	UPSA-B; SLOF	NA
Sabbag S et al., 2011	Assessing everyday functioning in schizophrenia: not all informants seem equally informative.	This study examined the convergence between self-reports on the part of people with schizophrenia (n = 193), whose real-world functioning was rated by a friend or relative (n = 154), or a high-contact clinician (n = 39) across six functional status rating scales.	Cross-sectional	Outpatient population, including community and residential care facilities	UPSA-B; SLOF; QLS; ILLS; SFS; LSP; SBS	NA

Study	Title	Study objective	Study design	Sample type	Measures reported	Intervention details
Bechi M et al., 2017	Exploring functioning in schizophrenia: predictors of functional capacity and real-world behaviour	To explore, through a comprehensive assessment, the interplay of clinical, neurocognitive and social cognitive domains, including empathy, as well as premorbid factors and their relative contributions in determining both functional capacity and real-life behavior in a sample of outpatients with chronic schizophrenia.	Cross-sectional	Community-dwelling outpatients	UPSA-B; QLS	NA
Czaja SJ et al., 2017	Assessing functional performance using computer-based simulations of everyday activities	To evaluate the feasibility of using a computer-based assessment battery for measuring the functional capacity of patients with schizophrenia.	Cross-sectional	Community-dwelling outpatients	UPSA-B; residential status (baseline); employment status (baseline)	NA
Keefe RSE et al., 2016	Validation of a computerized test of functional capacity	To 1) assess the validity, sensitivity, and reliability of the Virtual Reality Functional Capacity Assessment Tool (VRFCAT) as a measure of functional capacity; 2) determine the association between performance on the VRFCAT and performance on the MATRICS	Cross-sectional	Community-dwelling outpatients	UPSA-VIM; SLOF; employment status (baseline)	NA

Study	Title	Study objective	Study design	Sample type	Measures reported	Intervention details
		Consensus Cognitive Battery (MCCB); and 3) compare the metrics of the VRFCAT with the UCSD Performance-based Skills Assessment (UPSA).				
Koshikawa Y et al., 2016	The comparative effects of risperidone long-acting injection and paliperidone palmitate on social functioning in schizophrenia: a 6-month, open-label, randomized controlled pilot trial	To compare the effects of risperidone long-acting injection (RLAI) and paliperidone palmitate (PP) on non-acute-phase social functioning in patients with schizophrenia.	Randomized trial	Community-dwelling outpatients	UPSA-B; SFS; residential status (baseline)	Risperidone long-acting injection; paliperidone palmitate
Lee J et al., 2017	Deconstructing bipolar disorder and schizophrenia: a cross-diagnostic cluster analysis of cognitive phenotypes	To determine subgroups of individuals based on cognitive profile across clinical diagnoses, and to examine how the cluster membership is related to clinical diagnoses, with an assessment battery that covers perception, cognition, and social cognition on both auditory and visual modalities.	Cross-sectional	Community-dwelling outpatients	UPSA; RFS	NA

Study	Title	Study objective	Study design	Sample type	Measures reported	Intervention details
Moore RC et al., 2015	Improving the understanding of the link between cognition and functional capacity in schizophrenia and bipolar disorder	To determine whether a curvilinear relationship better approximates the association between cognitive functioning and functional capacity.	Cross-sectional	Community-dwelling outpatients	UPSA-B; living situation	NA
Ventura J et al., 2016	Cognitive Assessment Interview (CAI): validity as a co-primary measure of cognition across phases of schizophrenia	To further explore the validity of CAI by examining: 1) cross-sectional relationships with objective cognitive performance tests (MCCB), functional capacity (UPSA), and role functioning (RFS) in first-episode schizophrenia patients; 2) the effect sizes for CAI ratings in schizophrenia patients relative to those of healthy controls; and 3) the sensitivity of the CAI to changes in objective measures of cognition (MCCB).	Cross-sectional	Community-dwelling outpatients	UPSA; RFS	NA

Study	Title	Study objective	Study design	Sample type	Measures reported	Intervention details
Kumar S et al., 2016	An optimal combination of MCCB and CANTAB to assess functional capacity in older individuals with schizophrenia	To determine a minimal set of subtests across the MATRICS Consensus Cognitive Battery (MCCB) and the Cambridge Neuropsychological Test Automated Battery (CANTAB) that would be strongly associated with functional capacity in older patients with schizophrenia.	Cross-sectional	Community-dwelling outpatients	UPSA; living situation	NA

AR, affect recognition; BACS, Brief Assessment of Cognition in Schizophrenia; BADS, Behavioral Activation for Depression Scale; BFP, Brain Fitness Program; CAI, Cognitive Assessment Interview; CANTAB, Cambridge Neuropsychological Test Automated Battery; CT, cognitive training; CCT, Compensatory Cognitive Training; CNTRACS, Cognitive Neuroscience Test Reliability and Clinical Applications for Schizophrenia; COGS-2, Consortium on the Genetics of Schizophrenia case-control study; CVR, conventional vocational rehabilitation; C-UPSA, Computerized UPSA; DAFS, Direct Assessment of Functional Status; FAST, Functional Adaptation Skills Training; FC functional capacity; GAF, Global Assessment of Functioning; ILLS, Independent Living Skills Survey; ILS, Independent Living Scales; ILSS, Independent Living Skills Survey; IPS, Individual Placement and Support; LOS, late-onset schizophrenia; LSP, Life Skills Profile; MATRICS, Measurement and Treatment Research to Improve Cognition in Schizophrenia; MCCB, MATRICS Consensus Cognitive Battery; MoCA, Montreal Cognitive Assessment; MSIF, Multidimensional Scale of Independent Functioning; NA, not applicable; NCS, nonpsychiatric comparison subjects; NP, neuropsychological; PEDAL, *Programa de Entrenamiento para Desarrollo de Aptitudes para Latinos*; PP, paliperidone palmitate; PSP, Personal and Social Performance Scale; QLS, Quality of Life Scale; QOL, quality of life; QOLI, Quality of Life Interview; QWB, Quality of Well-being Scale; RCT, randomized controlled trials; RFS, Role Functioning Scale; RLAI, risperidone long-acting injection; SBS, Social Behavior Scale; SF-36, Medical Outcomes Survey–Short-form 36; SFS, Social Functioning Scale; SLOF, Specific Level of Function; SOF, Scale of Functioning; SP, standard pharmacotherapy; SSRI, selective serotonin reuptake inhibitors; STEP Skills Training and Empowerment Program; SZ, schizophrenia patients; UCSD, University of California at San Diego; UPSA, UCSD Performance-based Skills Assessment; UPSA-B, UPSA-Brief; UPSA-M, UPSA tablet/mobile application; UPSA-VIM, UPSA Validation of Intermediate Measures; UPSA-2, UPSA version 2; VRFCAT, Virtual Reality Functional Capacity Assessment Tool

Supplementary Table 4: STROBE statement checklist for included studies

Article	Q1	Q2	Q3	Q4	Q5	Q6	Q7	Q8	Q9	Q10	Q11	Q12	Q13	Q14	Q15	Q16	Q17	Q18	Q19	Q20	Q21	Q22
Abram et al. (2014)	No	Yes	Yes	Yes	Yes	Yes	Yes	Yes	No	No	Yes	Yes	No	Yes	Yes	Yes	Yes	Yes	Yes	Yes	No	Yes
Adelsky et al. (2011)	No	Yes	Yes	Yes	Yes	Yes	Yes	Yes	No	No	Yes	Yes	No	Yes	Yes	Yes	Yes	Yes	Yes	Yes	No	No
Alden et al. (2015)	Yes	Yes	Yes	Yes	Yes	Yes	Yes	Yes	No	No	Yes	Yes	No	Yes	Yes	Yes	Yes	Yes	Yes	Yes	No	Yes
Ammari et al. (2014)	No	Yes	Yes	Yes	Yes	Yes	Yes	Yes	No	No	Yes	No	No	Yes	Yes	Yes	Yes	Yes	Yes	Yes	Yes	No
Bechi et al. (2017	No	Yes	Yes	Yes	Yes	Yes	Yes	Yes	No	No	Yes	No	No	Yes	Yes	Yes	Yes	Yes	No	Yes	No	Yes
Bengoetxea et al. (2014)	No	Yes	Yes	Yes	Yes	Yes	Yes	Yes	No	No	Yes	Yes	No	Yes	Yes	Yes	Yes	Yes	Yes	Yes	Yes	No
Bowie et al. (2008)	Yes	Yes	Yes	Yes	Yes	Yes	Yes	Yes	Yes	No	Yes	Yes	No	Yes	Yes	Yes	Yes	Yes	Yes	Yes	No	Yes
Bowie et al. (2006)	Yes	Yes	Yes	Yes	Yes	Yes	Yes	Yes	Yes	No	Yes	Yes	No	Yes	Yes	Yes	Yes	Yes	Yes	Yes	No	Yes
Bowie et al. (2007)	Yes	Yes	Yes	Yes	Yes	Yes	Yes	Yes	Yes	No	Yes	Yes	No	Yes	Yes	Yes	Yes	Yes	Yes	Yes	No	Yes
Cardenas et al. (2013)	No	Yes	Yes	Yes	Yes	Yes	Yes	Yes	No	No	Yes	Yes	No	Yes	Yes	Yes	Yes	Yes	Yes	Yes	Yes	Yes
Czaja et al. (2017)	Yes	Yes	Yes	Yes	No	No	Yes	Yes	No	No	Yes	Yes	Yes	Yes	Yes	Yes	Yes	Yes	Yes	Yes	Yes	Yes
Depp et al. (2011)	No	Yes	Yes	Yes	Yes	Yes	Yes	Yes	No	No	Yes	Yes	No	Yes	Yes	Yes	Yes	Yes	Yes	Yes	Yes	Yes
Depp et al. (2016)	Yes	Yes	Yes	Yes	Yes	Yes	Yes	Yes	No	No	Yes	Yes	No	Yes	Yes	Yes	Yes	Yes	Yes	Yes	Yes	Yes
Durand et al. (2015)	Yes	Yes	Yes	Yes	Yes	Yes	Yes	Yes	No	No	Yes	No	No	Yes	Yes	Yes	Yes	Yes	Yes	Yes	No	Yes
Elliott et al. (2014)	Yes	Yes	Yes	Yes	Yes	Yes	Yes	Yes	No	No	Yes	Yes	No	Yes	Yes	Yes	Yes	Yes	Yes	Yes	No	Yes
Fiszdon et al. (2010)	No	Yes	Yes	Yes	Yes	Yes	Yes	Yes	No	No	Yes	Yes	No	Yes	Yes	Yes	Yes	Yes	Yes	Yes	Yes	Yes
Galderisi et al. (2014)	No	Yes	Yes	Yes	Yes	Yes	Yes	Yes	Yes	No	Yes	Yes	Yes	Yes	Yes	Yes	Yes	Yes	Yes	Yes	No	Yes

Article	Q1	Q2	Q3	Q4	Q5	Q6	Q7	Q8	Q9	Q10	Q11	Q12	Q13	Q14	Q15	Q16	Q17	Q18	Q19	Q20	Q21	Q22
Garcia-Portilla et al. (2013)	Yes	Yes	Yes	Yes	Yes	Yes	Yes	Yes	No	No	Yes	Yes	No	Yes	Yes	Yes	Yes	Yes	Yes	Yes	Yes	Yes
Gold et al. (2012)	No	Yes	Yes	Yes	Yes	No	Yes	Yes	No	No	Yes	Yes	No	Yes	Yes	Yes	Yes	Yes	Yes	Yes	No	Yes
Gould et al. (2012)	No	Yes	Yes	Yes	Yes	Yes	Yes	Yes	No	No	Yes	Yes	No	Yes	Yes	Yes	Yes	Yes	Yes	Yes	Yes	Yes
Green et al. (2008)	Yes	Yes	Yes	Yes	No	No	Yes	Yes	No	No	Yes	No	No	No	Yes	Yes	Yes	Yes	Yes	Yes	No	Yes
Green et al. (2011)	Yes	Yes	Yes	Yes	Yes	Yes	Yes	Yes	No	No	Yes	No	Yes	Yes	Yes	Yes	Yes	Yes	Yes	Yes	No	Yes
Gupta et al. (2012)	No	Yes	Yes	Yes	No	Yes	Yes	Yes	No	No	Yes	Yes	No	Yes	Yes	Yes	Yes	Yes	Yes	Yes	Yes	Yes
Harvey et al. (2009)	No	Yes	Yes	Yes	Yes	Yes	Yes	Yes	No	No	Yes	No	Yes	Yes	Yes	Yes	Yes	Yes	No	Yes	Yes	Yes
Harvey et al. (2009)	No	Yes	Yes	Yes	Yes	Yes	Yes	Yes	No	No	Yes	Yes	No	Yes	Yes	Yes	Yes	Yes	Yes	Yes	No	Yes
Heinrichs et al. (2010)	Yes	Yes	Yes	Yes	Yes	Yes	Yes	Yes	No	No	Yes	No	No	Yes	Yes	Yes	Yes	Yes	Yes	Yes	No	Yes
Heinrichs et al. (2006)	No	Yes	Yes	Yes	Yes	Yes	Yes	Yes	No	No	Yes	No	No	Yes	Yes	Yes	Yes	Yes	No	Yes	No	Yes
Helldin et al. (2012)	No	Yes	Yes	Yes	Yes	Yes	Yes	Yes	No	No	Yes	Yes	Yes	Yes	Yes	Yes	Yes	Yes	Yes	Yes	No	No
Ho et al. (2013)	No	Yes	Yes	Yes	No	Yes	Yes	Yes	Yes	No	Yes	Yes	No	Yes	Yes	Yes	Yes	Yes	Yes	Yes	No	Yes
Holshausen et al. (2014)	No	Yes	Yes	Yes	Yes	Yes	Yes	Yes	Yes	No	Yes	Yes	No	Yes	Yes	Yes	Yes	Yes	Yes	Yes	Yes	Yes
Jeste et al. (2005)	No	Yes	Yes	Yes	Yes	Yes	Yes	Yes	No	No	Yes	Yes	No	Yes	Yes	Yes	Yes	Yes	Yes	Yes	Yes	Yes
Kasckow et al. (2007)	No	Yes	Yes	Yes	Yes	Yes	Yes	Yes	No	No	Yes	Yes	Yes	Yes	Yes	Yes	Yes	Yes	Yes	Yes	No	Yes
Keefe et al. (2006)	No	Yes	Yes	No	Yes	Yes	Yes	Yes	No	No	No	No	No	Yes	Yes	Yes	Yes	Yes	No	Yes	No	Yes
Keefe et al. (2016)	Yes	Yes	Yes	Yes	No	No	Yes	Yes	No	No	Yes	Yes	Yes	Yes	Yes	Yes	Yes	Yes	Yes	Yes	No	Yes
Kim et al. (2015)	No	Yes	Yes	Yes	No	Yes	Yes	Yes	No	No	Yes	Yes	No	Yes	Yes	Yes	Yes	Yes	Yes	Yes	Yes	No

Article	Q1	Q2	Q3	Q4	Q5	Q6	Q7	Q8	Q9	Q10	Q11	Q12	Q13	Q14	Q15	Q16	Q17	Q18	Q19	Q20	Q21	Q22
Koshikawa et al. (2016)	Yes	Yes	Yes	Yes	Yes	Yes	Yes	Yes	No	No	Yes	Yes	Yes	Yes	Yes	Yes	Yes	Yes	Yes	Yes	Yes	No
Kumar et al. (2016)	No	Yes	Yes	Yes	No	Yes	Yes	Yes	No	No	Yes	Yes	No	Yes	Yes	Yes	Yes	Yes	Yes	Yes	No	Yes
Lee et al. (2015)	Yes	Yes	Yes	Yes	Yes	Yes	Yes	Yes	No	No	Yes	Yes	No	Yes	Yes	Yes	Yes	Yes	No	Yes	No	Yes
Lee et al. (2017)	Yes	Yes	Yes	Yes	No	No	No	Yes	No	No	Yes	No	Yes	Yes	Yes	Yes	Yes	Yes	Yes	Yes	No	Yes
Leifker et al. (2009)	No	Yes	Yes	Yes	Yes	Yes	Yes	Yes	No	No	Yes	Yes	No	Yes	Yes	Yes	Yes	Yes	Yes	Yes	No	Yes
Leung et al. (2008)	Yes	Yes	Yes	Yes	No	Yes	Yes	Yes	No	No	Yes	Yes	Yes	Yes	Yes	Yes	Yes	Yes	Yes	Yes	Yes	Yes
Light et al. (2005)	No	Yes	Yes	Yes	Yes	Yes	Yes	Yes	No	No	Yes	Yes	No	Yes	Yes	Yes	Yes	Yes	No	Yes	No	Yes
Light et al. (2012)	Yes	Yes	Yes	Yes	Yes	Yes	Yes	Yes	No	No	Yes	Yes	Yes	Yes	Yes	Yes	Yes	Yes	No	Yes	No	Yes
Mausbach et al. (2008)	Yes	Yes	Yes	Yes	Yes	Yes	Yes	Yes	No	No	Yes	Yes	No	Yes	Yes	Yes	Yes	Yes	Yes	Yes	No	Yes
Mausbach et al. (2011)	No	Yes	Yes	Yes	Yes	Yes	Yes	Yes	No	No	Yes	Yes	No	Yes	Yes	Yes	Yes	Yes	Yes	Yes	Yes	Yes
Mausbach et al. (2008)	No	Yes	Yes	Yes	Yes	Yes	Yes	Yes	No	No	Yes	Yes	Yes	Yes	Yes	Yes	Yes	Yes	Yes	Yes	Yes	Yes
Mausbach et al. (2010)	No	Yes	Yes	Yes	Yes	Yes	Yes	Yes	No	No	Yes	Yes	No	Yes	Yes	Yes	Yes	Yes	No	Yes	No	Yes
McClure et al. (2013)	No	Yes	Yes	Yes	Yes	Yes	Yes	Yes	No	No	Yes	Yes	No	Yes	Yes	Yes	Yes	Yes	Yes	Yes	No	Yes
Moore et al. (2015)	No	Yes	Yes	Yes	No	Yes	Yes	Yes	No	No	Yes	Yes	No	Yes	Yes	Yes	Yes	Yes	Yes	Yes	Yes	Yes
Moore et al. (2015)	No	Yes	Yes	Yes	No	No	Yes	Yes	No	No	Yes	Yes	Yes	Yes	Yes	Yes	Yes	Yes	Yes	Yes	Yes	Yes
Moore et al. (2013)	No	Yes	Yes	Yes	Yes	Yes	Yes	Yes	No	No	Yes	Yes	Yes	Yes	Yes	Yes	Yes	Yes	Yes	Yes	Yes	Yes
Muharib et al. (2014)	No	Yes	Yes	Yes	Yes	Yes	Yes	Yes	No	No	Yes	Yes	No	Yes	Yes	Yes	Yes	Yes	Yes	Yes	No	Yes
Murthy et al. (2012)	Yes	Yes	Yes	Yes	Yes	Yes	Yes	Yes	Yes	No	Yes	Yes	Yes	Yes	Yes	Yes	Yes	Yes	No	Yes	No	Yes

Article	Q1	Q2	Q3	Q4	Q5	Q6	Q7	Q8	Q9	Q10	Q11	Q12	Q13	Q14	Q15	Q16	Q17	Q18	Q19	Q20	Q21	Q22
Musso et al. (2014)	No	Yes	Yes	Yes	Yes	Yes	Yes	Yes	No	No	Yes	Yes	No	Yes	Yes	Yes	Yes	Yes	Yes	Yes	No	Yes
Narvaez et al. (2008)	No	Yes	Yes	Yes	No	Yes	Yes	Yes	No	No	Yes	Yes	No	Yes	Yes	Yes	Yes	Yes	Yes	Yes	Yes	No
Olsson et al. (2012)	No	Yes	Yes	Yes	Yes	Yes	Yes	Yes	No	No	Yes	Yes	No	Yes	Yes	Yes	Yes	Yes	Yes	Yes	No	Yes
Patterson et al. (2001)	No	Yes	Yes	Yes	Yes	Yes	Yes	Yes	No	No	Yes	Yes	No	Yes	Yes	Yes	Yes	Yes	Yes	Yes	Yes	Yes
Roseman et al. (2008)	Yes	Yes	Yes	Yes	No	Yes	Yes	Yes	No	No	Yes	Yes	No	Yes	Yes	Yes	Yes	Yes	Yes	Yes	Yes	Yes
Sabbag et al. (2011)	No	Yes	Yes	Yes	Yes	Yes	Yes	Yes	No	No	Yes	Yes	No	Yes	Yes	Yes	Yes	Yes	Yes	Yes	No	Yes
Sheffield et al. (2014)	No	Yes	Yes	Yes	Yes	Yes	Yes	Yes	No	No	Yes	Yes	No	Yes	Yes	Yes	Yes	Yes	Yes	Yes	Yes	Yes
Silverstein et al. (2011)	Yes	Yes	Yes	Yes	Yes	Yes	Yes	Yes	No	No	Yes	Yes	No	No	No	Yes	Yes	Yes	No	Yes	No	Yes
Smith et al. (2012)	No	Yes	Yes	Yes	Yes	Yes	Yes	Yes	No	No	Yes	Yes	No	Yes	Yes	Yes	Yes	Yes	Yes	Yes	Yes	Yes
Stergiopoulos et al. (2011)	No	Yes	Yes	Yes	Yes	Yes	Yes	Yes	No	No	Yes	Yes	Yes	Yes	Yes	Yes	Yes	Yes	Yes	Yes	Yes	Yes
Strassnig et al. (2015)	Yes	Yes	Yes	Yes	Yes	Yes	Yes	Yes	No	No	Yes	Yes	No	Yes	Yes	Yes	Yes	Yes	Yes	Yes	Yes	Yes
Thorp et al. (2012)	No	Yes	Yes	Yes	Yes	Yes	Yes	Yes	Yes	No	Yes	Yes	No	Yes	Yes	Yes	Yes	Yes	Yes	Yes	Yes	Yes
Twamley et al. (2011)	Yes	Yes	Yes	Yes	No	Yes	Yes	Yes	No	No	Yes	Yes	Yes	Yes	Yes	Yes	Yes	Yes	Yes	Yes	No	Yes
Twamley et al. (2002)	No	Yes	Yes	Yes	No	Yes	Yes	Yes	No	No	Yes	Yes	No	Yes	Yes	Yes	Yes	Yes	Yes	Yes	Yes	Yes
Twamley et al. (2012)	No	Yes	Yes	Yes	No	Yes	Yes	Yes	No	No	Yes	Yes	Yes	Yes	Yes	Yes	Yes	Yes	Yes	Yes	No	Yes
Twamley et al. (2012)	Yes	Yes	Yes	Yes	No	Yes	Yes	Yes	No	No	Yes	Yes	Yes	Yes	Yes	Yes	Yes	Yes	Yes	Yes	Yes	Yes
Ucok et al. (2012)	No	Yes	Yes	Yes	Yes	Yes	Yes	Yes	No	No	Yes	Yes	No	Yes	Yes	Yes	Yes	Yes	Yes	Yes	Yes	No
Vahia et al. (2010)	No	Yes	Yes	Yes	Yes	Yes	Yes	Yes	No	No	Yes	Yes	No	Yes	Yes	Yes	Yes	Yes	Yes	Yes	Yes	Yes
Ventura et al. (2016)	No	Yes	Yes	Yes	No	No	Yes	Yes	Yes	No	No	No	No	Yes	Yes	Yes	Yes	Yes	Yes	Yes	No	Yes

Article	Q1	Q2	Q3	Q4	Q5	Q6	Q7	Q8	Q9	Q10	Q11	Q12	Q13	Q14	Q15	Q16	Q17	Q18	Q19	Q20	Q21	Q22
Vesterager et al. (2012)	No	Yes	Yes	Yes	Yes	Yes	Yes	Yes	No	No	Yes	Yes	Yes	Yes	Yes	Yes	Yes	Yes	No	Yes	No	Yes

Supplementary Table 5: Studies reporting scores on the UPSA and the SLOF

Study	UPSA			SLOF			Correlations	
	Type	Mean	SD	Type	Mean	SD	rho	p-value
Bowie CR et al., 2006	UPSA	83.4	9.2	proxy	198.4	NA		
Bowie CR et al., 2007	UPSA	41.7	7.3	proxy	103.7	9.1		
Depp CA et al., 2011	UPSA	66.1	17.8	proxy	100.2	14.2		
Leung WW et al., 2008	UPSA	72.2	17.1	proxy	194.0	18.7		
Cardenas V et al., 2013	UPSA-B	54.0	21.5	proxy	98.8	15.7	0.22	<0.05
Depp CA et al., 2015	UPSA-B	53.5	20.6	proxy	97.8	18.2		
Gold JM et al., 2012	UPSA-B	77.0	13.4	proxy	175.9	25.4	0.39	<0.01
Gould F et al., 2012	UPSA-B	69.0	19.6	proxy	103.4	15.7		
Harvey PD, Helldin L et al., 2009[a]	UPSA-B	69.9	18.6	proxy	183.2	18.8	0.44	**[c]
Harvey PD, Helldin L et al., 2009[b]	UPSA-B	69.0	19.6	proxy	196.6	18.9	0.38	***[c]
Harvey PD, Keefe RS et al., 2009	UPSA-B	68.8	19.6	proxy	193.1	23.9		
Sheffield JM et al., 2014	UPSA-B	76.7	14.6	proxy	123.5	17.4		
Sabbag S et al., 2011	UPSA-B	76.7	13.1	proxy	172.1*	NA	0.19	NA
Galderisi S et al., 2014	UPSA-B	67.5	22.3	proxy	176.6	13.3		
Abram SV et al., 2014	UPSA-B	74.1	13.8	Self	126.8	12.8	0.4[d]	0.002
Alden EC et al., 2015	UPSA-B	74.0	10.2	Self	125.9	10.1		
Smith MJ et al., 2012	UPSA-B	76.2	13.8	Self	127.6	12.9	0.57	0.0001
Keefe RSE et al., 2016	UPSA-VIM	71	11.9	proxy	159.1[e]	6.1	0.25	<0.01

[a]Swedish cohort; [b]American cohort; [c]As reported by authors. Did not specify meaning; [d]Modified overall score, based on four out of six domains; [e]Modified overall score, based on three out of six domains.

Supplementary Table 6: Studies reporting scores on the UPSA and the GAF

Study	UPSA			GAF		Correlation	
	UPSA type	Mean	SD	Mean	SD	Rho	p-value
Bengoetxea E et al., 2014[a]	UPSA (Spanish)	47.5	20.4	55.5	6.4		
Bengoetxea E et al., 2014[b]	UPSA	58.7	18.5	52.7	5.3		
Bengoetxea E et al., 2014[c]	UPSA	53.3	19.8	52.2	4.8		
Fiszdon JM et al., 2010	UPSA	82.6	9.2	38.8	5.9		
Green MF et al., 2008	UPSA	86.1	11.3	59.2	9.3		
Light GA et al., 2005	UPSA	81.2	15.5	44.6	10.8	0.4	<0.05
Light GA et al., 2012	UPSA	78.3	14.3	44.0	6.4		
Garcia-Portilla MP et al., 2013	UPSA (Spanish)	70.2	18.1	57.4	17.2		
Lee J et al., 2014	UPSA-B	70.8	15.2	43.7	7.2		
Vesterager L et al., 2012	UPSA-B	77.5	13.3	46.6	10.5		
Olsson AK et al., 2012	UPSA-B (Swedish)	65.9*	n/a	47.6	8.01	0.3	0.01

[a] Monolingual Spanish speaking group; [b] English speaking latino group; [c] English speaking non-latino group; * Imputed value

Supplementary Table 7: Studies reporting scores on the UPSA and MSIF

Study	UPSA			MSIF		Correlation	
	UPSA type	Mean	SD	Mean	SD	Rho	p-value
Ammari N et al., 2014	UPSA	87.5	14.1	4.2	1.1		
Heinrichs RW et al., 2010	UPSA	83.0*	7.6*	4.4	1.1		
Heinrichs RW et al., 2006	UPSA	83.2	11.3	3.5	1.6	-0.4	<0.01
Muharib E et al., 2014	UPSA	78.7	10.8	3.9	1.2		
Silverstein SM et al., 2011	UPSA	79.7	11.0	4.4	1.3		
Sheffield JM et al., 2014	UPSA-B	76.7	14.6	3.7	1.3		

* Estimated value

Supplementary Table 8: Studies reporting UPSA scores and baseline residential status

Study	Living situation (%)					UPSA		
	Living independently	Assisted living	Restricted living	Institutionalized	Other	UPSA type	Mean	SD
Koshikawa Y et al., 2016	100	0	0	0	0	UPSA-B	70.9	12.9
Helldin L et al., 2011	98	0	0	2	0	UPSA-B	62.8	NR
Depp CA et al., 2015	98	0	0	0	2	UPSA-B	53.5	20.6
Harvey PD et al., 2009	95	0	5	0	0	UPSA-B	69.9	18.6
Moore RC et al., 2013	91	10	0	0	0	UPSA	72.9	17
Olsson AK et al., 2012	90	8	0	0	2	UPSA-B	65.9	NR
Mausbach BT et al., 2011	89	11	0	0	0	UPSA-B	77.5	17.7
Mausbach BT et al., 2010	88	13	0	0	0	UPSA-B	75.6	20.8
Vesterager L et al., 2012	87	13	0	0	0	UPSA-B	77.5	13.3
Narvaez JM et al., 2008	82	14	0	0	4	UPSA	83.3	10
Twamley EW et al., 2012	81	19	0	0	0	UPSA	83.8	9.2
Twamley EW et al., 2011	80	20	0	0	0	UPSA	82.7	9.4
Sabbag S et al., 2011	79	22	0	0	0	UPSA-B	76.7	13.1
Garcia-Portilla et al., 2013	77	0	0	14	9	UPSA	70.2	18.1
Kumar S et al., 2016	77	17	0	2	0	UPSA	72.8	15.8
Light GA et al., 2005	66	44	0	0	0	UPSA	81.2	15.5
Leung WW et al., 2008	64	0	36	0	0	UPSA	72.2	17.1
Czaja SJ et al., 2017	58	0	0	32	10	UPSA-B	70.2	17.3
Holshausen K et al., 2013	56	44	0	0	0	UPSA	76.9	9.6
Murthy NV et al., 2012	56	42	0	0	0	UPSA-2	80	1.3
Kasckow J et al., 2007	54	46	0	0	0	UPSA	76.3	15.2
Moore RC et al., 2015	51	NR	NR	NR	NR	UPSA-B	77.3	17.9
McClure MM et al., 2013	44	56	0	0	0	UPSA-B	78.3	11.5
Thorp SR et al., 2012	41	54	3	1	4	UPSA	73.1	17.9
Bowie CR et al., 2008	41	59	0	0	0	UPSA	70.6	19.2
Jeste ND et al., 2005	38	63	0	0	0	UPSA	60.7	19.9

Study	Living situation (%)					UPSA		
	Living independently	Assisted living	Restricted living	Institutionalized	Other	UPSA type	Mean	SD
Twamley EW et al., 2002	38	59	3	1	0	UPSA	66.4	23.1
Mausbach BT et al., 2008	31	69	0	0	0	UPSA-B	54.1	23.5
Depp CA et al., 2011	30	70	0	0	0	UPSA	66.1	17.8
Patterson TL et al., 2001	24	76	0	0	0	UPSA	58.8	27.1
Mausbach BT et al., 2008	23	77	0	0	0	UPSA	66.4	19.2
Moore RC et al., 2015	21	NR	NR	NR	NR	UPSA-B	53	20.7
Moore RC et al., 2015	10	90	0	0	0	UPSA	74.7	11.9
Stergiopoulos V et al., 2011	0	0	0	41	59	UPSA	70.2	15
Keefe RS et al., 2006	0	0	0	100	0	UPSA	80.9	10
Adelsky MB et al., 2010	0	0	0	100	0	UPSA-B	35.4	22.4

Abbreviations: NR - Not reported.

Supplementary Table 9: Studies reporting UPSA and baseline employment status

	Employment status (%)			UPSA		
Study	Not Employed	Employed[a]	Other[b]	UPSA type	Mean	SD
Olsson AK et al., 2012	15	12	73	UPSA-B (Swedish)	65.9	0
Garcia-Portilla MP et al., 2013	27	9	65	UPSA (Spanish)	70.2	18.1
Bowie CR et al., 2008	59	41	0	UPSA	70.6	19.2
Mausbach BT et al., 2011	61	32	7	UPSA-B	77.5	17.7
Mausbach BT et al., 2010	63	28	10	UPSA-B	75.6	20.8
Leung WW et al., 2008	69	20	11	UPSA	72.2	17.1
Helldin L et al., 2011	70	27	3	UPSA-B	62.8	4.8
Ammari N et al., 2014	71	29	0	UPSA	87.5	14.1
Galderisi S et al., 2014	71	29	0	UPSA-B	67.5	22.3
Czaja SJ et al., 2017	72	9	19	UPSA-B	70.2	17.3
Ucok A et al., 2012	72	0	28	UPSA-B	12.6[c]	4.6
Harvey PD et al., 2009	81	19	0	UPSA-B	69.9	18.6
Murthy NV et al., 2012	84	11	4	UPSA-2	80	1.3
Sabbag S et al., 2011	84	13	3	UPSA-B	76.7	13.1
Keefe RSE et al., 2016	85	NR	NR	UPSA-VIM	71.0	11.9
Muharib E et al., 2014	87	13	0	UPSA	78.7	10.8
Narvaez JM et al., 2008	94	6	0	UPSA	83.3	10
Stergiopoulos V et al., 2011	94	6	0	UPSA	70.2	15
Depp CA et al., 2011	97	3	0	UPSA	66.1	17.8

[a] Full-time or part-time; [b] Retired, volunteer, student, sheltered employment, disability; [c] Only raw score reported
Abbreviations: NR - Not reported.
